# Long non-coding RNA enhances SARS-CoV-2-mediated apoptosis through epigenetic repression of angiotensin-converting enzyme 2

**DOI:** 10.1016/j.jbc.2025.110812

**Published:** 2025-10-13

**Authors:** Weijie Liao, Tian Zhang, Jiaxing Cao, Songmao Wang, Yaou Zhang

**Affiliations:** 1 Department of Hematology and Oncology, Shenzhen University General Hospital, International Cancer Center, Shenzhen University, Shenzhen, Guangdong, China; 2 Tsinghua Shenzhen International Graduate School, Tsinghua University, Shenzhen, Guangdong, China; 3 Department of Intelligent Computing Research, Peng Cheng Laboratory, Shenzhen, Guangdong, China; 4 Department of Computer Technology, Changjiang Institute of Technology, Wuhan, Hubei, China

**Keywords:** SARS-CoV-2, HIF-1α, EPB41L4A-AS1, ACE2, apoptosis

## Abstract

Despite its critical role in organ protection, angiotensin-converting enzyme 2 (ACE2) is paradoxically downregulated during SARS-CoV-2 infection, contributing to multi-organ dysfunction in COVID-19. The mechanisms driving this loss, particularly those mediated by long non-coding RNAs (lncRNAs), are unknown. Through transcriptomic analysis, we identified that the lncRNA EPB41L4A-AS1 (EAS1) is consistently upregulated upon infection by multiple coronaviruses, including SARS-CoV-2, SARS-CoV, MERS-CoV, and HCoV-229E. Mechanistically, SARS-CoV-2 infection activates HIF-1α, which binds to an enhancer element within the EAS1 locus to drive its expression. Subsequently, EAS1 recruits the acetyltransferase GCN5 to catalyze the acetylation of the transcriptional coactivator PGC1β. This modification disrupts the interaction between PGC1β and the transcription factor PPARγ, thereby impairing PPARγ-mediated transactivation of the ACE2 gene and leading to its transcriptional repression. Functionally, we demonstrate that this EAS1-mediated suppression of ACE2 exacerbates cellular apoptosis induced by TNF-α and hypoxia, two key pathological features of severe COVID-19. Our study unveils a novel epigenetic pathway through which SARS-CoV-2 dysregulates ACE2 expression and promotes cellular damage. Given the persistent threat of recurrent coronavirus spillovers, the conserved upregulation of EAS1 across distinct coronavirus genera suggests it may represent a potential therapeutic target for mitigating organ injury in COVID-19. Furthermore, our findings that the EAS1-ACE2 axis mediates apoptosis in response to hypoxia and cytokine signaling warrant future investigation into its role in other pathological contexts beyond viral infection.

The repeated emergence of highly pathogenic beta-coronaviruses, such as Severe Acute Respiratory Syndrome Coronavirus (SARS-CoV) and Middle East Respiratory Syndrome Coronavirus (MERS-CoV), has posed significant threats to global public health over the past 2 decades (1). The ongoing pandemic caused by SARS-CoV-2, another member of this genus, has led to unprecedented morbidity, mortality, and socioeconomic disruption worldwide (2). While vaccination campaigns have effectively mitigated the severity of the disease and enabled a return to societal normalcy (3, 4), the pathogenic mechanisms underlying Coronavirus Disease 2019 (COVID-19), particularly in severe cases presenting with multi-organ dysfunction, remain incompletely elucidated. Critically, the recurrent spillover of coronaviruses from animal reservoirs presents a persistent threat, with future outbreaks likely to continue inflicting devastating impacts on human health. This looming risk underscores the urgent need to elucidate conserved, pan-coronaviral pathogenic mechanisms, which are essential for developing broad-spectrum therapeutic strategies against both current and future coronavirus threats.

A critical step in SARS-CoV-2 pathogenesis is viral entry, which is mediated by the binding of the viral Spike protein's receptor-binding domain (RBD) to the host receptor angiotensin-converting enzyme 2 (ACE2) on the surface of target cells (5, 6, 7). ACE2 is widely expressed across diverse human tissues, including the lung, heart, kidney, liver, and intestines (8), which aligns with the systemic nature of SARS-CoV-2 infection and suggests that cells with higher ACE2 expression levels are more susceptible to viral invasion. Beyond its role as a viral receptor, ACE2 is a key component of the renin-angiotensin system (RAS), where it counterbalances ACE activity by converting angiotensin II (Ang II) into angiotensin 1 to 7, thereby exerting protective effects including vasodilation, anti-inflammatory, and anti-apoptotic functions (9). Consequently, the dysregulation of ACE2 expression has been implicated in the pathophysiology of various cardiovascular, renal, and pulmonary diseases (10). A hallmark of SARS-CoV-2 infection is a significant reduction in ACE2 expression (11, 12, 13, 14). Proposed mechanisms for this downregulation include Spike protein-induced internalization and lysosomal degradation (11, 12), viral non-structural protein 1 (NSP1)-mediated suppression of ACE2 translation or mRNA stability (13), and epigenetic repression driven by inflammatory cytokines such as TNFα (14). This loss of ACE2 is hypothesized to deprive tissues of its protective functions, potentially exacerbating organ injury in patients with COVID-19. However, the precise regulatory pathways governing ACE2 depletion during infection are not fully defined.

Long non-coding RNAs (lncRNAs) have emerged as potent regulators of gene expression in various physiological and pathological processes, including viral infections (15, 16). High-throughput studies have identified numerous lncRNAs that are dysregulated following SARS-CoV-2 infection (17, 18), yet the functional consequences and mechanistic roles of most of these alterations remain largely unexplored. For instance, while lncRNAs like DANCR and NEAT1 have been associated with the inflammatory response to SARS-CoV-2 (19), a direct causal relationship has not been firmly established. This gap in knowledge prompts the hypothesis that specific lncRNAs may play crucial roles in mediating the host's pathological response, including the dysregulation of ACE2.

In this study, we aimed to identify and characterize lncRNAs that are consistently modulated by coronavirus infection and to investigate their potential role in regulating ACE2 expression and COVID-19 pathogenesis. We focused on the lncRNA EAS1, a molecule previously studied by our group in other disease contexts (20, 21, 22), due to its conserved upregulation across infections with multiple coronaviruses. We sought to delineate the mechanism by which SARS-CoV-2 induces EAS1 expression, how EAS1 in turn epigenetically represses ACE2 transcription, and the functional impact of this axis on cellular apoptosis. Our findings unveil a novel regulatory pathway that connects viral infection to ACE2 suppression and tissue damage, highlighting EAS1 as a potential therapeutic target for coronavirus-related diseases.

## Results

### SARS-CoV-2 and other coronaviruses consistently upregulate EAS1

**Figure 1:**
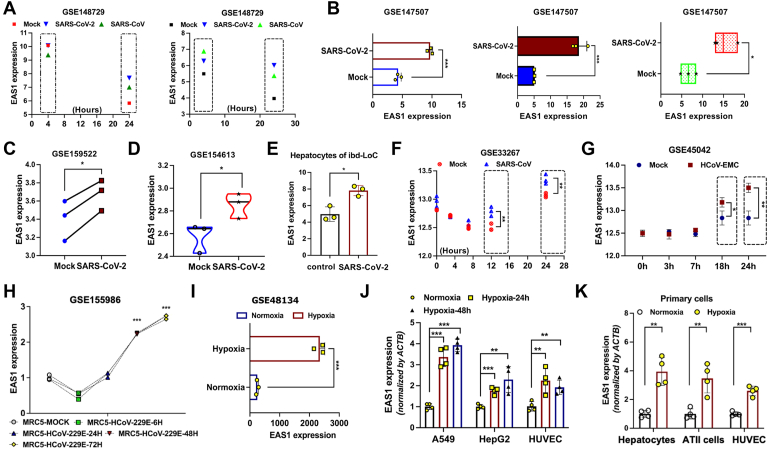
**SARS-CoV-2 and other coronaviruses consistently upregulate EAS1.***A*, RNA-seq analysis of EAS1 expression in Caco-2 cells infected with SARS-CoV-2 and Calu-3 cells infected with SARS-CoV at the indicated time points (n = 2 biologically independent samples). *B*, EAS1 expression in Calu-3, A549, and ACE2-overexpressing A549 (ACE2-A549) cells 24 h post-infection (hpi) with SARS-CoV-2 (n = 3). *C*, EAS1 levels in ACE2-A549 cells infected with SARS-CoV-2 (MOI = 0.1) for 24 h (n = 3). *D*, EAS1 expression in ACE2-A549 cells 24 hpi with SARS-CoV-2 (USA-WA1/2020) (n = 3). *E*, EAS1 levels in a blood vessel-liver organoid co-culture model 4 days post SARS-CoV-2 infection (n = 3). *F*, Time course of EAS1 expression in Calu-3 cells infected with SARS-CoV (Delta ORF6-mutant, MOI = 5) (n = 3). *G*, EAS1 expression in Calu-3 2B4 cells infected with MERS-CoV (HCoV-EMC) (n = 3). *H*, EAS1 levels in MRC5 cells infected with HCoV-229E (n = 3). *I*, EAS1 expression in A549 cells under hypoxia (1%O_2_) for 42 h (n = 3). *J*, qPCR analysis of EAS1 in HepG2, A549, and immortalized HUVEC cells under hypoxia (1% O_2_) for 24 and 48 h (n = 4 biologically independent experiments, each performed in triplicate). *K*, qPCR analysis of EAS1 in primary human hepatocytes, AT II cells, and HUVECs from four distinct donors under hypoxia (1% O_2_) for 24 h (n = 3 independent experiments, each in triplicate). Data are mean ± SD. *p* values were determined by a two-tailed paired Student's *t* test, ∗∗∗*p* < 0.001, ∗∗*p* < 0.01, ∗*p* < 0.05.

To identify lncRNAs involved in coronavirus pathogenesis, we analyzed host transcriptomic responses. We discovered that the lncRNA EAS1 was significantly upregulated 24 h post-infection with SARS-CoV-2 across multiple models, including Calu-3, A549, ACE2-overexpressing A549 (ACE2-A549) cells and blood vessel-liver organoid (Fig. 1, *A*–*E*). We next investigated whether EAS1 induction was a common feature of coronavirus infections. Indeed, infection with other β-coronaviruses, including SARS-CoV and MERS-CoV, similarly led to a significant increase in EAS1 expression at 24 h post-infection (Fig. 1, *A*, *F* and *G*). Notably, even infection with the α-coronavirus HCoV-229E induced EAS1 upregulation (Fig. 1*H*), suggesting a conserved role for EAS1 in the host response to diverse coronaviruses.

Given that severe COVID-19 is frequently associated with hypoxia, we tested if hypoxic conditions alone could stimulate EAS1 expression. Hypoxia treatment markedly upregulated EAS1 expression not only in A549, HepG2, and immortalized HUVEC cells (Fig. 1, *I* and *J*), but also in primary hepatocytes, alveolar type II (AT II) cells, and primary HUVECs (Fig. 1*K*), identifying hypoxia as a potential driver of EAS1 overexpression during infection.

### HIF-1α mediates the induction of EAS1 by SARS-CoV-2

**Figure 2:**
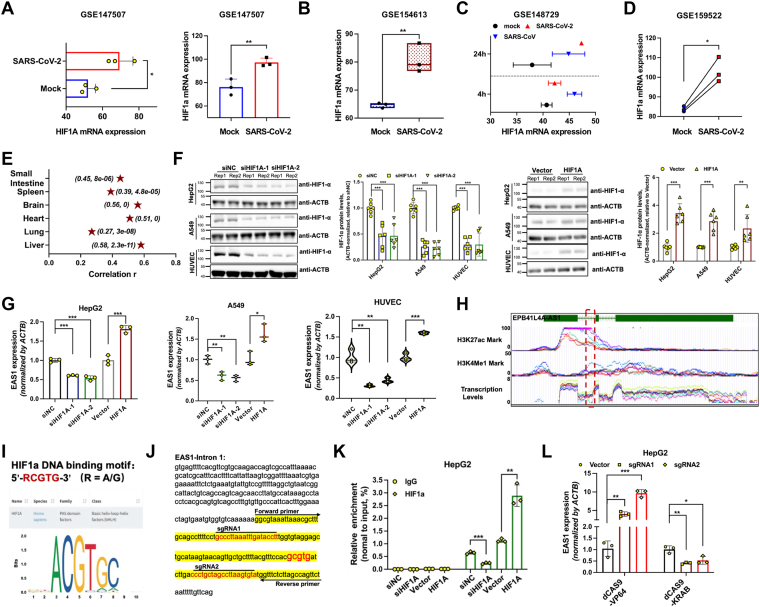
**HIF-1α mediates the induction of EAS1 by SARS-CoV-2.** (*A*) HIF1A mRNA levels in A549 and ACE2-A549 cells 24 hpi with SARS-CoV-2 (n = 3); (*B*) HIF1A expression in ACE2-A549 cells infected with SARS-CoV-2 (USA-WA1/2020) for 24 h (n = 3); (*C*) HIF1A levels in Calu-3 cells infected with SARS-CoV-2 or SARS-CoV (n = 2); (*D*) HIF1A expression in ACE2-A549 cells infected with SARS-CoV-2 (MOI = 0.1) for 24 h (n = 3); (*E*) Pearson correlation analysis of HIF1A and EAS1 expression across human tissues from the GTEx database, results are shown as (Pearson Correlation Coefficient, *p*-value); (*F*) Western blot analysis of HIF-1α protein levels after knockdown (KD) or overexpression (OE) in HepG2, A549, and immortalized HUVEC cells (n = 3 independent experiments, each in twice); (*G*) qPCR analysis of EAS1 expression (n = 3, each in triplicate); (*H*) Enrichment levels of H3K27ac and H3K4me1 at EAS1 genomic loci and transcriptional activity analysis, data were extracted from the UCSC Genome Browser (GRCh38/hg38 assembly); (*I*) DNA-binding motif of HIF1A from the JASPAR database; (*J*) Schematic of the HIF1α-binding site within the first intron of EAS1, indicating locations for ChIP-qPCR primers and CRISPRi and CRISPRa sgRNA; (*K*) ChIP-qPCR analysis of HIF1α binding at the EAS1 enhancer following HIF1A KD or OE (n = 3, each in triplicate); (*L*) qPCR analysis of EAS1 expression after targeted epigenetic repression or activation (dCas9-VP64 or dCas9-KRAB) of the enhancer region (n = 3, each in triplicate). Data are mean ± SD. *p* values were determined by a two-tailed paired Student's *t* test, ∗∗∗*p* < 0.001, ∗∗*p* < 0.01, ∗*p* < 0.05.

Since hypoxia induces HIF-1α, and SARS-CoV-2 infection can mimic hypoxic conditions, we hypothesized that HIF-1α might be responsible for EAS1 upregulation. We found that SARS-CoV-2 infection itself significantly increased HIF-1α mRNA levels (Fig. 2, *A*–*D*). Analysis of GTEx datasets revealed a strong positive correlation between HIF-1A and EAS1 expression in human tissues (Figs. 2*E* and S1*A*).

Functional experiments confirmed the regulatory relationship between HIF-1α and EAS1, as knockdown of HIF-1α decreased EAS1 expression, whereas overexpression of HIF-1α increased EAS1 levels (Figs. 2, *F*, *G* and S1, *B* and *C*).To mechanistically link HIF-1α to EAS1 transcription, we interrogated the EAS1 genomic locus and identified a putative enhancer region in its first intron, marked by active histone modifications (H3K27ac, H3K4me1) and a HIF-1α binding motif (Fig. 2, *H* and *I*). Chromatin immunoprecipitation (ChIP) assays confirmed HIF-1α binding to this site, which was abrogated by HIF-1α knockdown and enhanced by its overexpression (Fig. 2, *J* and *K*). Finally, CRISPR-based interference (CRISPRi) and activation (CRISPRa) of this enhancer region specifically decreased and increased EAS1 expression, respectively (Fig. 2, *J* and *L*). Together, these data establish that SARS-CoV-2-induced HIF-1α directly binds to an enhancer within the EAS1 gene to drive its transcription.

### EAS1 functions as a negative regulator of ACE2 expression

**Figure 3:**
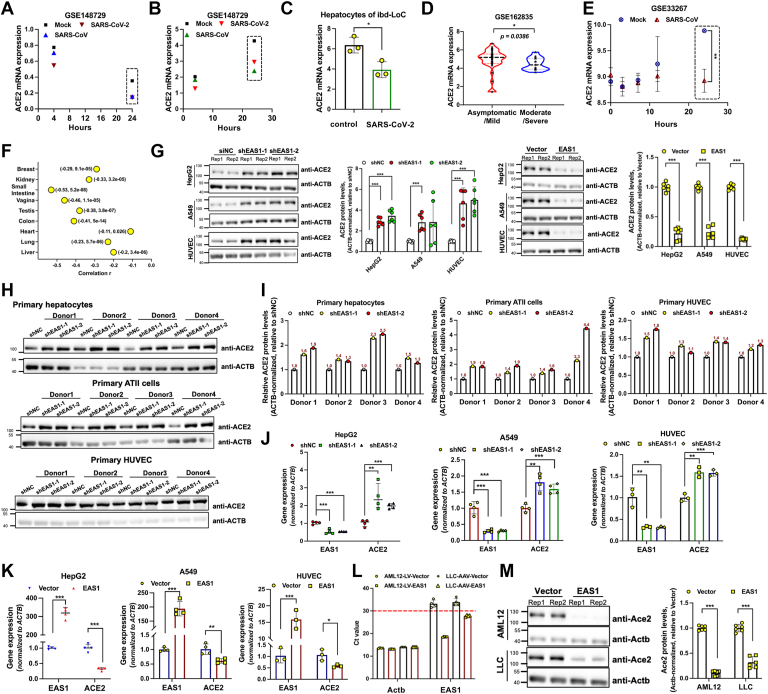
**EAS1 functions as a negative regulator of ACE2 expression.***A-B*, ACE2 expression in Caco-2 (*A*) and Calu-3 (*B*) cells infected with SARS-CoV-2 or SARS-CoV (n = 2). *C*, ACE2 mRNA levels in the liver organoid model from Fig. 1*E* (n = 3). *D*, ACE2 mRNA levels in patients with mild (n = 37) *versus* severe (n = 13) COVID-19, *p* value was determined by a Mann-Whitney U test. *E*, Time course of ACE2 expression in Calu-3 cells infected with SARS-CoV (Delta ORF6-mutant, MOI = 5) (n = 3). *F*, Peason correlation analysis of ACE2 and EAS1 expression using RNAseq data of different tissues from GTEx database, results are shown as (Pearson Correlation Coefficient, *p*-value). *G*, Western blot analysis of ACE2 protein levels after EAS1 KD or OE for 48 h (n = 3, each in twice). *H-I*, Western blot of ACE2 in primary cells from 12 donors transduced with EAS1 shRNA for 48 h (representative of n = 1 experiment); (*J*-*K*) qPCR quantification of ACE2 mRNA levels following EAS1 KD or OE in various cell lines (n = 3-4, each in triplicate). *L*, qPCR verification of human EAS1 expression in mouse AML12 (lentiviral) and LLC (AAV-DJ) cells (n = 4, each in triplicate). *M*, Western blot analysis of mouse Ace2 protein levels in AML12 and LLC cells overexpressing human EAS1 for 48 h (n = 3, each in twice). For all panels, data are mean ± SD. Statistical significance was determined by a two-tailed paired Student's *t* test unless noted otherwise, ∗∗∗*p* < 0.001, ∗∗*p* < 0.01, ∗*p* < 0.05.

SARS-CoV-2 infection entails downregulation of its cognate receptor, ACE2. We confirmed that infection with SARS-CoV-2 led to a significant reduction in ACE2 expression at 24 h (Fig. 3, *A* and *B*). This downregulation was also evident in blood vessel-liver organoid (Fig. 3*C*) and clinical samples, with lower ACE2 levels observed in late-stage COVID-19 patients compared to those in the early stage (Fig. 3*D*). A downregulation of ACE2 was also confirmed after SARS-CoV infection (Fig. 3, *A*, *B* and *E*).

Given the temporal correlation between EAS1 upregulation and ACE2 downregulation, we investigated a potential causal link. We found a significant negative correlation between EAS1 and ACE2 expression across normal human tissues (Figs. 3*F* and S2, *A*–*C*). Functionally, shRNA-mediated knockdown of EAS1 elevated ACE2 protein levels in HepG2, A549, and immortalized HUVEC cells, while its overexpression reduced them (Figs. 3*G* and S2, *D* and *E*). Consistent effects were observed in primary hepatocytes, AT II cells, and primary HUVECs (Fig. 3, *H* and *I*). Moreover, EAS1 knockdown increased ACE2 mRNA levels across multiple cell types, including HepG2, A549, immortalized HUVEC, U251, and NP69 cells. Conversely, EAS1 overexpression decreased ACE2 mRNA levels (Figs. 3, *J*, *K* and S2*F*). Importantly, this regulatory axis is conserved across species, as overexpression of human EAS1 in mouse hepatocyte (AML12) and lung carcinoma (LLC) cells significantly reduced mouse Ace2 protein levels (Figs. 3, *L*, *M* and S2*G*). These results demonstrate that EAS1 is a potent repressor of ACE2.

### EAS1 represses ACE2 transcription *via* the GCN5-PGC1β-PPARγ axis

**Figure 4:**
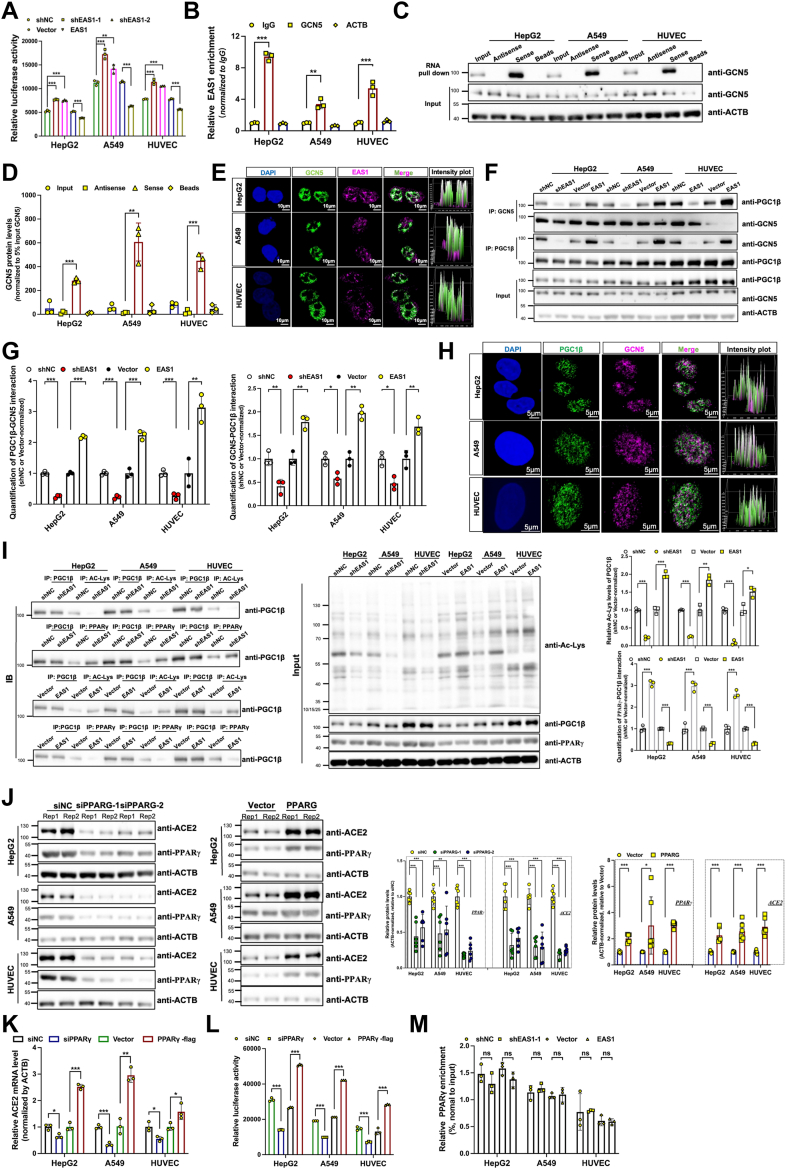
**EAS1 represses ACE2 transcription *via* the GCN5-PGC1β-PPARγ axis.***A* ,luciferase reporter assay measuring ACE2 promoter activity after EAS1 KD or OE (n = 3, each in triplicate). *B*, RIP-qPCR analysis of the endogenous EAS1-GCN5 interaction. IgG served as a negative control (n = 3, each in triplicate). *C-D*, RNA pull-down assay using biotinylated sense or antisense EAS1 RNA, followed by Western blot for GCN5 (n = 3). *E*, RNA FISH combined with IF analysis of EAS1 (*magenta*) and GCN5 (*green*) co-localization. Nuclei are stained with DAPI (*blue*). Scale bar = 10 μm. *F-G*, Co-IP analysis of endogenous PGC1β-GCN5 binding after EAS1 KD or OE (n = 3). *H*, IF staining showing co-localization of PGC1β (*green*) and GCN5 (*magenta*), scale bars = 5 μm. *I*, Co-IP analysis of PGC1β acetylation (Ac-K) and PGC1β-PPARγ interaction after EAS1 KD or OE (n = 3); *J*, Western blot analysis of PPARγ and ACE2 protein levels after PPARγ KD or OE for 48 h (n = 3, each in twice); *K*, qPCR analysis of ACE2 mRNA after PPARγ KD or OE for 48 h (n = 3, each in triplicate); *L*, Luciferase reporter assay of ACE2 promoter activity after PPARγ KD or OE for 48 h (n = 3, each in triplicate); *M*, ChIP-qPCR analysis of PPARγ enrichment at the ACE2 promoter after EAS1 KD or OE (n = 3, each in triplicate). Data are mean ± SD. Statistical significance was determined by a two-tailed paired Student's *t* test, ∗∗∗*p* < 0.001, ∗∗*p* < 0.01, ∗*p* < 0.05.

We next explored the mechanism by which EAS1 suppresses ACE2. EAS1 knockdown enhanced, while its overexpression inhibited, ACE2 promoter activity (Fig. 4*A*), indicating transcriptional regulation. We previously reported that EAS1 can recruit the acetyltransferase GCN5. RIP, RNA pull-down, and FISH/IF co-localization (secondary antibody alone as negative control staining) assays confirmed an interaction between EAS1 and GCN5 (Figs. 4, *B*–*E* and S3, *A* and *B*).

We further found that EAS1 enhances the interaction between GCN5 and PGC1β, as evidenced by a reduction in their binding upon EAS1 knockdown and an increase upon EAS1 overexpression (Figs. 4, *F*–*H* and S3, *C* and *D*). EAS1 expression enhanced the acetylation of PGC1β (Figs. 4*I* and S3*E*). Since PGC1β is a known coactivator of PPARγ, a transcription factor reported to bind the ACE2 promoter, we validated that PPARγ is a positive regulator of ACE2 transcription (Figs. 4, *J*–*L* and S3*F*). Crucially, we discovered that EAS1 disrupted PGC1β′s interaction with PPARγ (Fig. 4*I*), without affecting PPARγ′s binding to the ACE2 promoter (Fig. 4*M*). Since PGC1β′s transcriptional activation activity is impaired by acetylation, we hypothesized that EAS1-mediated PGC1β acetylation is the mechanism underlying this disrupted interaction. This disruption ultimately impairs PPARγ-mediated transactivation of ACE2. Thus, EAS1 acts as an epigenetic scaffold that recruits GCN5 to acetylate PGC1β, thereby dissociating the PGC1β-PPARγ complex and silencing ACE2 transcription.

### Inhibition of EAS1 attenuates SARS-CoV-2 infection-related apoptosis

**Figure 5:**
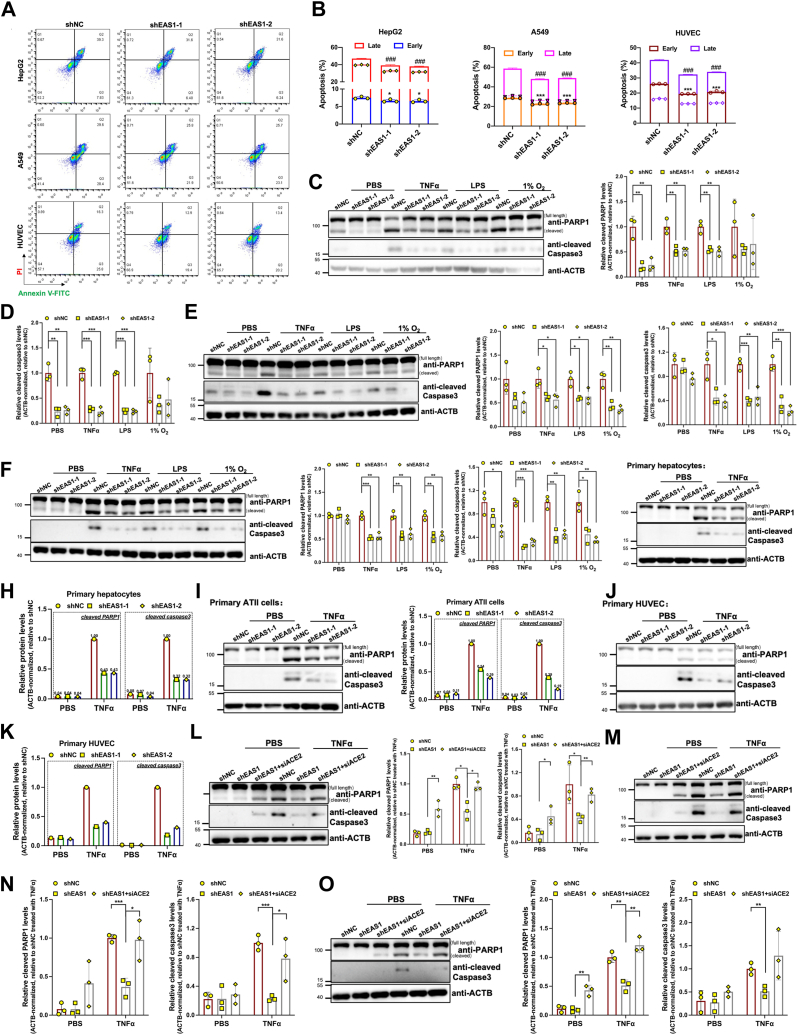
**Inhibition of EAS1 attenuates SARS-CoV-2 infection-related apoptosis.***A-B*, flow cytometry analysis of apoptosis (Annexin V/PI staining) in HepG2, A549, and immortalized HUVEC cells treated with TNFα (HepG2-20ng, A549/immortalized HUVEC-10ng) for 24 h after EAS1 KD (n = 3). *C-F*, Western blot analysis of cleaved PARP1 and cleaved caspase 3 in EAS1 KD cells treated with TNFα (HepG2-20ng, A549/immortalized HUVEC-10ng), LPS (20 μg), or hypoxia (1%O_2_) for 24 h (n = 3). *G-K*, Western blot analysis of apoptosis markers in primary human hepatocytes, AT II cells, and primary HUVECs with EAS1 KD under TNFα treatment for 24 h (representative of n = 1 experiment). *L-O*, Western blot analysis of apoptosis markers in EAS1 KD cells upon concomitant ACE2 KD and TNFα treatment (n = 3). Data are mean ± SD. Statistical significance was determined by a two-tailed paired Student's *t* test, ∗∗∗*p* < 0.001, ∗∗*p* < 0.01, ∗*p* < 0.05.

Severe COVID-19 is characterized by cytokine release and hypoxia, both potent inducers of apoptosis. We confirmed that SARS-CoV-2 infection upregulates the key cytokine TNFα *in vitro* and in lung tissues from deceased patients (Fig. S4, *A*–*D*). We established that 10 ng/ml TNFα was an optimal dose for inducing apoptosis in A549 and immortalized HUVEC cells (Fig. S4*E*). To investigate whether EAS1 contributes to this pathology, we knocked down EAS1 under TNFα-induced pro-apoptotic conditions. Annexin V-FITC/PI staining showed that EAS1 suppression significantly reduced apoptosis (Fig. 5, *A* and *B*). Further studies under hypoxia, TNFα, and LPS stimulation confirmed that EAS1 knockdown markedly attenuated apoptosis in HepG2, A549, and immortalized HUVEC cells (Figs. 5, *C*–*F* and S4, *F*–*H*). Moreover, EAS1 knockdown also alleviated TNFα-induced apoptosis in primary hepatocytes, AT II cells, and primary HUVECs (Fig. 5, *G*–*K*). Most importantly, the anti-apoptotic effect of EAS1 knockdown under TNFα treatment was completely abolished when ACE2 was co-silenced (Figs. 5, *L*–*O* and S4, *I*–*K*). This experiment demonstrates that ACE2 is the essential downstream mediator through which EAS1 promotes apoptosis. These findings position the EAS1-ACE2 axis as a potential regulator of cellular damage in the pathological milieu of severe COVID-19.

## Discussion

**Figure 6:**
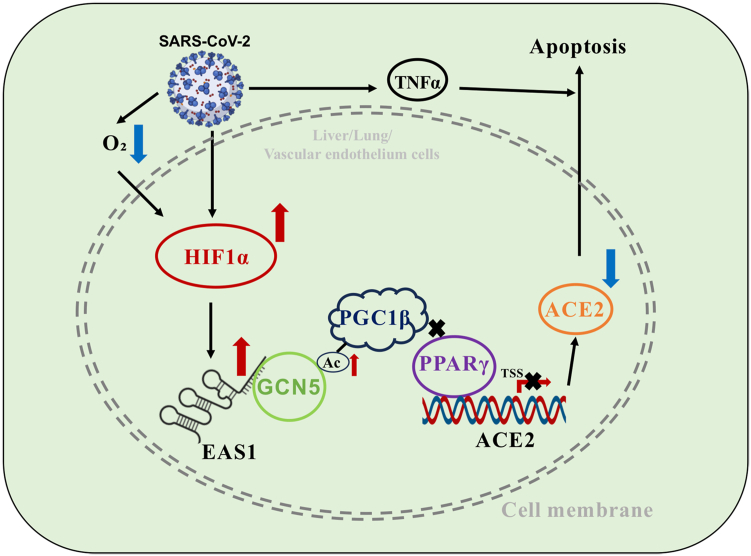
**Summary of the proposed model for EAS1-mediated repression of ACE2 and promotion of apoptosis during SARS-CoV-2 infection.** SARS-CoV-2 infection activates HIF-1α, which transactivates the lncRNA EAS1. EAS1 recruits the acetyltransferase GCN5, which acetylates the transcriptional coactivator PGC1β. Acetylation disrupts the PGC1β-PPARγ complex, leading to transcriptional repression of ACE2. The subsequent downregulation of ACE2 sensitizes cells to apoptosis triggered by cytokine storm (TNFα) and hypoxia, contributing to multi-organ damage in severe COVID-19.

In this study, we identify the lncRNA EAS1 as a potential epigenetic regulator that mediates SARS-CoV-2-induced ACE2 downregulation and subsequent cellular apoptosis. We delineate a novel pathway wherein SARS-CoV-2 infection activates HIF-1α, which transactivates EAS1 by binding to an intronic enhancer. EAS1, in turn, recruits the acetyltransferase GCN5 to catalyze PGC1β acetylation, disrupting the PGC1β-PPARγ complex and ultimately repressing ACE2 transcription. This axis functionally contributes to apoptosis under conditions mimicking severe COVID-19, such as cytokine storm and hypoxia (Fig. 6). Crucially, the conservation of EAS1 upregulation across multiple coronaviruses underscores its potential as a broad-spectrum therapeutic target.

Our findings provide a potential mechanistic explanation for the well-documented yet poorly understood phenomenon of ACE2 downregulation following SARS-CoV-2 infection. While previous reports attributed ACE2 loss to mechanisms like viral entry-mediated internalization, NSP1-driven mRNA degradation, or cytokine-induced epigenetic silencing (11, 12, 13, 14), our work unveils a distinct lncRNA-dependent pathway. The EAS1-GCN5-PGC1β-PPARγ axis operates at the transcriptional level by modulating coactivator-function, a strategy that may allow the virus to more permanently suppress host protective genes. The finding that human EAS1 protein can suppress mouse Ace2 expression underscores the relevance of animal models for further mechanistic and therapeutic studies.

The role of EAS1 extends beyond ACE2 regulation to directly impact cell fate decisions critical to COVID-19 pathology. Severe disease is characterized by a vicious cycle of viral replication, inflammation, and hypoxia, leading to widespread tissue damage (23, 24). We demonstrate that EAS1 is not only induced by hypoxia but also potently sensitizes cells to apoptosis triggered by TNFα and other stressors. The fact that the anti-apoptotic effect of EAS1 knockdown is entirely dependent on ACE2 upregulation solidifies a direct link between the loss of this protective receptor and the execution of cell death. This dual function of EAS1, depleting ACE2 while priming cells for apoptosis, may explain the strong correlation between ACE2 deficiency, inflammatory markers, and organ dysfunction in advanced COVID-19.

Beyond the mechanism elucidated here, the observed inverse correlation between EAS1 upregulation and ACE2 downregulation could potentially be explained by alternative pathways. For instance, it is possible that other transcription factors or epigenetic regulators, responsive to the inflammatory milieu of COVID-19, might also contribute to the suppression of ACE2 expression independently or in concert with the EAS1-GCN5 axis. Additionally, while our data firmly establish a post-translational mechanism *via* PGC1β acetylation, we cannot rule out the possibility that EAS1 might also influence ACE2 mRNA stability through sequestering microRNAs or RNA-binding proteins. The potential existence of these parallel mechanisms does not diminish the significance of our findings but rather highlights the complexity of host-virus interactions and underscores the need for further investigation to fully delineate the regulatory network controlling ACE2 expression during viral infection.

Our work reveals a lncRNA-mediated epigenetic pathway for ACE2 silencing. This adds to the growing recognition of non-coding RNAs in COVID-19 pathogenesis, where miRNAs have been prominently featured. Circulating miR-1-3p levels predict severity and mortality by enhancing endothelial dysfunction (25), while miR-142 (targeting TIM-1) and miR-24 (targeting Neuropilin-1) regulate alternative viral entry factors (26, 27). Distinct miRNA signatures, including miR-27a-5p and miR-21-5p, are linked to mild disease (28), and miRNA dysregulation persists in long COVID (29). Furthermore, network-based analyses of COVID-19/asthma comorbidity have identified key hub genes and regulatory miRNAs, highlighting the complex interplay between host factors and non-coding RNAs in disease susceptibility (30). Robust profiling of these signals relies on proper normalization, as demonstrated by the identification of miR-205-3p as a stable reference (31).

We acknowledge several limitations that should be considered when interpreting our findings. First, the primary data are derived from *in vitro* cell culture models. While we utilized multiple cell lines and including primary human cells, it is important to note that the sample size (n) for these primary cell experiments, though derived from multiple donors, remains limited due to the inherent challenges of procuring and culturing these tissues. While the consistent trends we observed are encouraging, future studies with larger cohorts will be essential to solidify these findings and better account for potential donor-to-donor variability. Second, the lack of *in vivo* validation in an animal model of COVID-19 remains a significant gap. Due to the low evolutionary conservation of lncRNAs, future studies employing EAS1-humanized mouse models infected with SARS-CoV-2 are essential to confirm the pathophysiological relevance of this axis *in vivo*. Thirdly, our study only included a limited number of strains analysis, including USA-WA1/2020. Due to the continuous mutation of SARS-CoV-2 and the emergence of numerous variants, our findings may not be representative of all viral strains. It is critical to examine whether different variants modulate the HIF-1α-EAS1-ACE2 axis with different efficiencies, which could influence disease progression. Fourthly, the relative contributions of hypoxia *versus* direct viral factors or inflammatory cytokines to EAS1 induction *in vivo* remain to be dissected. This could be addressed in future work by comparing EAS1 expression in infected models with and without supplemental oxygen or cytokine blockade. Finally, as EPB41L4A-AS1 is an antisense lncRNA to the EPB41L4A protein-coding gene, its effects may be partially mediated through regulating the expression of EPB41L4A. While our study focused on its trans-regulatory role *via* the GCN5-PGC1β axis, we cannot rule out potential cis-effects on its neighboring gene. The potential role of the EPB41L4A protein itself in SARS-CoV-2 infection or COVID-19 pathogenesis remains an important question for future investigations.

Despite these limitations, our study establishes a novel regulatory pathway that connects viral infection to ACE2 suppression and cellular damage. The consistent upregulation of EAS1 across multiple coronavirus genera (including SARS-CoV, MERS-CoV, and HCoV-229E) suggests that this mechanism is not unique to SARS-CoV-2 and may therefore represent a potential host-directed target worthy of investigation for mitigating organ injury in COVID-19 and, potentially, in diseases caused by other coronaviruses. Furthermore, our demonstration that the EAS1-ACE2 axis mediates apoptosis in response to hypoxia and TNFα, key drivers of COVID-19 pathology, provides a mechanistic basis for its contribution to disease severity. The conserved nature of this mechanism warrants further exploration of its role in the pathogenesis of future coronavirus outbreaks.

## Experimental procedures

### Reagents

The detailed information of reagents, including puromycin, Lipopolysaccharides (LPS), TNFα and polybrene, used in this study were provided in Table S1.

### Cell lines and culture conditions

Human Hepatoma cell line HepG2 (ATCC HB-8065) and human lung cancer cell line A549 (ATCC CCL-185) were purchased from American Type Culture Collection (ATCC) and maintained in Dulbecco's Modified Eagle Medium (DMEM, Gibco, 11995065) containing 10% fetal bovine serum (FBS, Biowest, S1810). Human immortalized NP69 cell line was purchased from Shunran Biology (Shanghai, China) and cultured in DMEM with 10% FBS. Human immortalized HUVEC (SCSP-5330), U251 (SCSP-559), murine hepatocyte AML12 (SCSP-550) and mouse lung adenocarcinoma LLC (SCSP-5252) cell lines were obtained from National Collection of Authenticated Cell Cultures (Shanghai, China). Immortalized HUVEC, U251 and LLC cells were maintained in DMEM with 10% FBS, and AML12 cells were maintained in DMEM/F-12 medium (1:1, Gibco, 11330032/11039021) with 10% FBS, 1% ITS (Sigma, I3146) and 40 ng/ml Dexamethasone (Sigma, 50-02-2). All the above cells were cultured at 37 °C in a humidified atmosphere with 5% CO_2_. The short tandem repeat (STR) profiling of all cell lines was performed by the supplier and were routinely screened for *mycoplasma* contamination using a PCR-based kit (TransGen, FM321). All experiments were performed with cells within 25 passages.

### Generation of stable cell lines

For stable knockdown of EAS1, two distinct short hairpin RNAs (shRNAs) targeting EAS1 and a non-targeting control (NC) shRNA were cloned into lentiviral interference vector LV-2N (pGLVU6/Puro, GenePharma, C06002), and packaged into lentivirus by GenePharma. Lentivirus were used to infect target cells in the presence of 5 μg/ml polybrene (MedChemExpress, HY-112735). After 12 h, the medium was replaced with fresh complete medium. 48 h post-infection, cells were selected with 2 μg/ml puromycin (Gibco, A1113803). When all untransfected cells had died, the surviving cells that had been successfully transduced were considered as the stable pools. For transient knockdown, cells were analyzed 48 to 72 h after lentiviral infection without selection. For overexpression, EAS1 transcript was clone into a lentiviral LV6 (EF-1a/Puro, GenePharma) or an adeno-associated viral vector (AAV-DJ, GenePharma). Human cells lines and murine AML12/LLC cells were transduced to generate human EAS1 overexpressing cells.

### Gene silencing and transfection

For transient gene knockdown, cells were transfected with 50 nM specific siRNAs or a non-targeting control siRNA (GenePharma) using Lipofectamine 3000 (Invitrogen, L3000015) according to the manufacturer’s protocol followed by 48 h of incubation. For CRISPRi and CRISPRa, sgRNAs were designed using Crispr-ERA tool (http://crispr-era.stanford.edu/index.jsp). The sgRNA targets were as follows: sgRNA1-gcccttaaatttgatacctt and sgRNA2-ccctgctagccttaagtgta. pLX-sgRNAs, dCAS9-VP64_GFP, and pHR-SFFV-dCAS9-BFP-KRAB vectors were synthesized by Youbio (Changsha, China). For plasmid transfections, cells at 60 to 80% confluence were transfected with 2 μg (6-well dish) or 8 μg (10-cm dish) of plasmid DNA using Lipofectamine 3000 and analyzed 48 h later. The sequences of siRNAs were shown in Table S2.

### Primary cell isolation

Human liver tissue, lung tissue, and umbilical cord tissue samples (n = 4 each) were obtained for primary cell isolation, following approval by the Ethics Committee of Shenzhen University General Hospital in 2023 (Approval No. KYLL-20230311). The studies in this work abide by the Declaration of Helsinki principles. Primary hepatocytes were isolated from liver tissue, alveolar type II epithelial cells (AT II) from lung tissue, and human umbilical vein endothelial cells (HUVECs) from umbilical cords. Detailed isolation protocols are described below:

Isolation of Primary Hepatocytes: Liver tissues were trimmed and minced into 1 to 2 mm^3^ fragments. A two-step collagenase perfusion method was used for digestion. First, tissues were digested in calcium- and magnesium-free Hanks' Balanced Salt Solution (HBSS) containing 0.5 mM EGTA with shaking at 37 °C for 15 min. After washing with PBS, digestion was continued using HBSS (with calcium and magnesium ions) containing 0.1% (w/v) type IV collagenase and 5 mM CaCl_2_ with shaking at 37 °C for 30 min. The reaction was stopped by adding an equal volume of DMEM high-glucose medium supplemented with 10% fetal bovine serum (FBS). The mixture was gently pipetted, filtered sequentially through 100 μm and 70 μm cell strainers, and centrifuged at 50*g* for 5 min at 4 °C to collect cells.

Isolation of Alveolar Type II Epithelial Cells (AT II): Lung tissues were rinsed with ice-cold DPBS, and airways and blood vessels were removed. Tissues were minced into ∼1 mm^3^ pieces and digested with a mixture of 0.25% trypsin and 0.1% collagenase I in DPBS with shaking at 37 °C for 45 to 60 min. The digestion was terminated by adding an equal volume of DMEM/F12 medium containing 10% FBS. The cell suspension was filtered through 100 μm and 40 μm cell strainers and centrifuged at 150*g* for 5 min at 4 °C. To enrich AT II cells, the cell pellet was resuspended and plated on culture flasks coated with type I rat tail collagen (5 μg/cm^2^) for 1 h at 37 °C and 5% CO_2_. Fibroblasts were removed by differential attachment. Non-adherent cells were collected and transferred to new collagen-coated plates for further culture. The medium was replaced after 24 to 48 h.

Isolation of Human Umbilical Vein Endothelial Cells (HUVECs): The umbilical vein was rinsed with sterile DPBS until no blood remained, followed by perfusion with 0.1% (w/v) type II collagenase solution and incubation at 37 °C for 15 min. The digest was collected and mixed with an equal volume of Endothelial Cell Medium (ScienCell, #1001) containing 20% FBS to terminate the reaction. After filtration, cells were collected by centrifugation at 200*g* for 5 min at room temperature. The cell pellet was resuspended in complete Endothelial Cell Medium (supplemented with endothelial cell growth supplement) and seeded onto culture vessels pre-coated with 0.1% gelatin. After 24 h of culture at 37 °C and 5% CO_2_, the medium was replaced to remove non-adherent cells.

### Bioinformatics analysis

RNAseq count data of GSE148729 (32), GSE147507 (33), GSE159522 (34), GSE154613 (35), GSE33267 (36), GSE45042 (37, 38), GSE48134 (39), GSE162835 (40) and GSE193330 (41) were retrieved from the Gene Expression Omnibus (GEO) database. Count data were normalized and converted to Fragments Per Kilobase of transcript per Million mapped reads (FPKM) values using the *R* software. For comparative analysis with normal tissue expression patterns, matched RNA-seq data from the Genotype-Tissue Expression (GTEx) project were analyzed through the Gene Expression Profiling Interactive Analysis 2 (GEPIA2) platform (http://gepia.cancer-pku.cn) (42), where Pearson correlation coefficients were calculated using log2(TPM+1) transformed expression values. Histone modification profiles (H3K27ac and H3K4me1) and transcriptional regulatory data for the EAS1 locus were obtained from the Encyclopedia of DNA Elements (ENCODE) Consortium. These datasets were visualized and analyzed using the UCSC Genome Browser (GRCh38/hg38 assembly) with integrated tools for chromatin state annotation and transcription factor binding site prediction.

### Reverse transcription quantitative real-time PCR (RT-qPCR)

Total RNA was extracted from cells using TRIzol reagent ((Invitrogen, 15596026) following the manufacturer's protocol and precipitated with isopropanol and finally purified with 75% ethanol. RNA concentration and purity were measured using a NanoDrop 2000 (Thermo Fisher Scientific, ND-2000). 1 μg of total RNA was reverse-transcribed into cDNA using ReverTra Ace qPCR RT Master Mix with gDNA Remover kit (TOYOBO, FSQ-301). RT-qPCR was carried out using SYBR Green Realtime PCR Master Mix (TOYOBO, QPK-201). The thermocycling conditions were: 95 °C for 60 s, followed by 40 cycles of 95 °C for 15 s and 60 °C for 45 s. A melt curve analysis was performed at the end of each run to confirm amplification specificity. Gene relative expression levels were calculated using 2^-ΔΔCt^, normalizing against ACTB. Each sample was analyzed from at least three independent biological experiments, each in triplicate unless noted otherwise. The primer sequences are listed in Table S3, and the expected size of the amplicon in bp, amplicon Sanger sequencing, the annealing temperatures, GenBank Accession numbers, and the verification of primer specificity (confirmed by *in silico* BLAST analysis) and amplicon identity (confirmed by Sanger sequencing of the PCR products) were shown in Supporting Information-2. The raw RT-qPCR data are shown in Supporting Information-3.

### Western blotting

Cells were collected and lysed in cell protein lysis buffer on ice for more than 30 min, the components of the lysate are as follows: 4M urea, 50 mM Tris-HCl (pH = 8.0), 1% Triton X-100 and protease inhibitor (Roche, 04693132001). Lysates were centrifuged at 12,000*g* for 15 min at 4 °C. Supernatant protein concentrations were determined using Bradford Protein Assay Kit (Beyotime, P0006) and separated by SDS-PAGE on 7.5%/10%/12.5% gels and transferred to nitrocellulose membranes (Millipore, HATF00010). Membranes were blocked with 5% non-fat milk in TBST for 1 h at room temperature and then incubated with primary antibodies overnight at 4 °C. After washing, membranes were incubated with species-matched horseradish peroxidase (HRP)-conjugated secondary antibodies (1:5000, abcam) for 1 h at room temperature. Protein bands were visualized using Tanon High-sig ECL Western Blotting Substrate (Tanon, 180-5001) and image using iBright 750 Imaging System (Thermo Fisher Scientific). Densitometric analysis was performed using ImageJ software. The antibodies used for detection were presented in Table S4, and the uncropped gels were shown in Supporting Information-4.

### Luciferase reporter assay

The ACE2 promoter region (−2000 bp relative to the transcription start site (TSS)) was cloned into the pGL3-Basic vector. HEK293T cells were seeded in 12-well plates and co-transfected with 1 μg pGL3-ACE2-promoter along with either 50 nM siEAS1/siPPARγ or 1 μg EAS1/PPARγ overexpression plasmid using Lipofectamine 3000. Cells were lysed 48 h post-transfection, and firefly luciferase activity was measured using a luciferase assay system (Promega, e500). Relative luminescence units were normalized to total protein concentration. Results are presented as relative luciferase activity (fold-change) from at least three independent experiments.

### RNA pull-down assay

We performed RNA pull-down assays using the biotinylated RNA Pull-Down Kit (FI8702-24T) from FITGENE. The sense and antisense sequences of EAS1 were individually cloned into the pcDNA3.1 vector containing a T7 promoter. Following plasmid linearization with restriction enzymes, *in vitro* transcription was conducted using the MEGAscript Kit (Ambion, #1334), and the RNA products were 3′-end labeled with biotin. For each reaction, 3 μg of biotinylated RNA was denatured at 95 °C for 3 min and immediately chilled on ice for 1 min for renaturation, then incubated with streptavidin magnetic beads at room temperature for 30 min with constant mixing. In parallel, cellular lysates were prepared from 2 × 10^7^ to 4 × 10^7^ cells, and the supernatant proteins were incubated with the RNA-bead complexes at 4 °C for 2 to 4 h with rotation. After extensive washing, the bound proteins were eluted with 1 × SDS-PAGE loading buffer and subsequently analyzed by Western blotting.

### RNA immunoprecipitation (RIP)

Cells were washed with DEPC treated PBS for three times, crosslinked using 37% formaldehyde for 10 min, stopped crosslinked with glycine and lysed in polysome lysis buffer (10 mM KCl, 10 mM Hepes, 1 mM DTT, 5 mM MgCl2, 0.5% NP-40, 100 U/ml RNase inhibitor and proteinase inhibitor). Cells were sonicated at low power with 5 s for 3 cycles and then supernatant was collected. The lysis was incubated with primary antibody on a rotator overnight at 4 °C. The antibody-cell lysate was next incubated with 80 μl Dynabeads protein G for another 4 h. Then, Dynabeads-antibody-cell lysate was washed with 600 μl polysome lysis buffer for four times and polysome lysis buffer containing 1M urea for another four times. Crosslinking was reversed by adding 100 μl polysome lysis buffer containing 30 μg proteinase K and 0.1% SDS at 55 °C for 30 min. RNA was extracted by 100ul of phenol-chloroform-isoamyl alcohol (25:24:1) and precipitated with sodium acetate, ethanol and glycogen at −80 °C overnight or more time. Further, RNA was purified by pre-chilled 75% ethanol and then used for the next qPCR detection. The primer sequences used for RIP-qPCR are shown in Table S3. The results were normalized to IgG and calculated from three independent biological duplications.

### Co-immunoprecipitation (Co-IP)

Protein A/G magnetic beads (50 μl, MCE HY-K0202) were equilibrated in PBS and incubated with 4 μg each of antibodies against GCN5, PGC-1β, Acetyl-Lysine (Ac-Lys), and PPARγ at room temperature for 30 min with gentle rotation (20 rpm) to form antibody-bead complexes. Beads were then washed twice with PBS to remove unbound antibodies. Concurrently, cells were lysed in ice-cold RIPA buffer (Beyotime, P0013C) supplemented with 1 × protease inhibitor cocktail and 1 mM PMSF. Lysates were vortexed briefly and incubated on ice for 30 min with periodic mixing, followed by centrifugation at 12,000*g* for 15 min at 4 °C to collect supernatants. Protein concentrations were determined by BCA assay (Beyotime, P0012) according to manufacturer's instructions, using BSA as standard. Equal amounts of protein lysates were pre-cleared with 20 μl protein A/G beads for 1 h at 4 °C before incubation with antibody-bead complexes. The pre-cleared lysates were incubated with antibody-bead complexes at 4 °C for 2 h with constant rotation. The complexes were then washed five times with 1 ml ice-cold PBS containing 0.25% Tween-20. After final wash, bound proteins were eluted by boiling in 40 μl 1 × SDS-PAGE loading buffer at 95 °C for 10 min. Eluates were separated and analyzed by Western blotting.

### Chromatin immunoprecipitation (ChIP)

Cells were washed and crosslinked using 1% formaldehyde for 15 mins at room temperature. Glycine was added to a final concentration of 0.125 M to quench crosslinking. The cells then were harvested with ChIP lysis buffer, the components of the lysate are as follows: 1% Triton X-100, 50 mM Tris-HCl (pH = 8.0), 150 mM NaCl, 5 mM EDTA, 0.1% deoxycholate and protease inhibitor cocktail. After sonicating (30% power, 18 cycles, 5 s on, 25 s off) of cells, the supernatant was immunoprecipitated with 2 μg primary antibody or IgG control antibody for 2 h at 4 °C. Further, the cell lysate-antibody was incubated with 25 μl Dynabeads protein G for another 2 h at 4 °C. After extensive washing, the DNA fragments were reversed crosslinking by proteinase K and purified. Enrichment of specific genomic regions was analyzed by qPCR using primers listed in Table S3.

### RNA fluorescence *in situ* hybridization (FISH) and immunofluorescence (IF)

For RNA FISH, the subcellular localization of EAS1 was determined using the Biosearch Technologies Stellaris RNA FISH kit according to the manufacturer’s protocol. Stellaris FISH probes labeled with Quasar 570 were designed against the full-length EAS1 transcript. Cells were cultured in 12 well cell culture plate with cover glass inserts and fixed using fixation buffer. Then cells were washed, permeabilized in 0.1% Triton X-100 in PBS (DEPC), and incubated with Hybridization Buffer containing probe overnight at 37 °C. Finally, the nucleus was stained by DAPI and the localization of EPB41L4A-AS1 was imaged using.

For IF, cells were seeded on coverslips and allowed to adhere, followed by fixation with 4% paraformaldehyde for 15 min at room temperature (RT). Permeabilization was performed with 0.25% Triton X-100 for 10 min, and non-specific binding was blocked with 5% BSA for 1 h at RT. Primary antibodies (rabbit anti-PGC-1β, 1:100; mouse anti-GCN5, 1:200) were applied for 1 h at RT, followed by appropriate secondary antibodies (Alexa Fluor 488-conjugated goat anti-rabbit IgG, 1:500; Alexa Fluor 647-conjugated goat anti-mouse IgG, 1:500), which could be accessible to the color blind, for 1 h at RT in the dark. Nuclei were counterstained with DAPI (1 μg/ml) for 5 min, and coverslips were mounted with anti-fade mounting medium. All images were acquired using a Nikon A1R HD25 confocal microscopy with a 60 × oil immersion objective. Quantitative analysis of three-dimensional fluorescence intensity profiles of nuclear co-localization of PGC-1β (Green) and GCN5 (Magenta) was performed using ImageJ software.

### Flow cytometry for apoptosis

Apoptosis was assessed using the Annexin V-FITC/PI Apoptosis Detection Kit (TransGen Biotech, Cat# FA101-01). Briefly, 1 × 10^6^ cells from each experimental group were collected and washed twice with ice-cold 1 × PBS. The cell pellet was resuspended in 195 μl of Annexin V-FITC binding buffer, followed by gentle mixing with 5 μl of Annexin V-FITC staining solution and incubation for 10 min at room temperature in the dark. After centrifugation, the supernatant was discarded, and cells were resuspended in 190 μl of fresh binding buffer. Subsequently, 10 μl of PI staining solution (20 μg/ml) was added, and the cells were incubated on ice for 5 min in the dark. Following staining, 300 μl of ice-cold PBS was added, and samples were analyzed within 1 h using a flow cytometer (BD, CytoFLEX). Data were processed using FlowJo software, with apoptotic populations quantified as follows: viable cells (Annexin V^−^/PI^−^), early apoptotic cells (Annexin V^+^/PI^−^), and late apoptotic/necrotic cells (Annexin V^+^/PI^+^).

### Statistical analysis

Results were shown as means ± standard deviation (SD) as indicated unless noted otherwise and analyzed by two-tailed Student’s *t* test or Mann-Whitney U test. Statistically significant was considered if the *p* value < 0.05 (∗*p* < 0.05, ∗∗*p* < 0.01, and ∗∗∗*p* < 0.001). All the experiments were performed at least three times unless noted otherwise and data plots were constructed by GraphPad Prism 8.0 or 10.0 software.

## Data availability

All the data are available in the article and Supplementary Files, or available from the authors upon request.

## Supporting information

This article contains supporting information.

## Conflict of interest

The authors declare that they have no conflicts of interest with the contents of this article.

## References

[1] Korber B., Fischer W.M., Gnanakaran S., Yoon H., Theiler J., Abfalterer W. (2020). Tracking changes in SARS-CoV-2 spike: evidence that D614G increases infectivity of the COVID-19 virus. Cell.

[2] Wu Y., Ho W., Huang Y., Jin D.Y., Li S., Liu S.L. (2020). SARS-CoV-2 is an appropriate name for the new coronavirus. Lancet.

[3] Yin W., Xu Y., Xu P., Cao X., Wu C., Gu C. (2022). Structures of the omicron spike trimer with ACE2 and an anti-Omicron antibody. Science.

[4] ACTIV-3/Therapeutics for Inpatients with COVID-19 (TICO) Study Group (2022). Efficacy and safety of two neutralising monoclonal antibody therapies, sotrovimab and BRII-196 plus BRII-198, for adults hospitalised with COVID-19 (TICO): a randomised controlled trial. Lancet Infect. Dis..

[5] Lan J., Ge J., Yu J., Shan S., Zhou H., Fan S. (2020). Structure of the SARS-CoV-2 spike receptor-binding domain bound to the ACE2 receptor. Nature.

[6] Wang Q., Zhang Y., Wu L., Niu S., Song C., Zhang Z. (2020). Structural and functional basis of SARS-CoV-2 entry by using human ACE2. Cell.

[7] Yan R., Zhang Y., Li Y., Xia L., Guo Y., Zhou Q. (2020). Structural basis for the recognition of SARS-CoV-2 by full-length human ACE2. Science.

[8] Li M.Y., Li L., Zhang Y., Wang X.S. (2020). Expression of the SARS-CoV-2 cell receptor gene ACE2 in a wide variety of human tissues. Infect. Dis. Poverty.

[9] Kuba K., Imai Y., Ohto-Nakanishi T., Penninger J.M. (2010). Trilogy of ACE2: a peptidase in the renin-angiotensin system, a SARS receptor, and a partner for amino acid transporters. Pharmacol. Ther..

[10] Gheblawi M., Wang K., Viveiros A., Nguyen Q., Zhong J.C., Turner A.J. (2020). Angiotensin-Converting enzyme 2: SARS-CoV-2 receptor and regulator of the renin-angiotensin System: celebrating the 20th anniversary of the discovery of ACE2. Circ. Res..

[11] Hoffmann M., Kleine-Weber H., Schroeder S. (2020). SARS-CoV-2 cell entry depends on ACE2 and TMPRSS2 and is blocked by a clinically proven protease inhibitor. Cell.

[12] Lei Y., Zhang J., Schiavon C.R., He M., Chen L., Shen H. (2021). SARS-CoV-2 spike protein impairs endothelial function via downregulation of ACE 2. Circ. Res..

[13] Thoms M., Buschauer R., Ameismeier M., Koepke L., Denk T., Hirschenberger M. (2020). Structural basis for translational shutdown and immune evasion by the Nsp1 protein of SARS-CoV-2. Science.

[14] Kenney A.D., Zani A., Kawahara J., Eddy A.C., Wang X.L., Mahesh K.C. (2023). Interferon-induced transmembrane protein 3 (IFITM3) limits lethality of SARS-CoV-2 in mice. EMBO Rep..

[15] Wang K.C., Chang H.Y. (2011). Molecular mechanisms of long noncoding RNAs. Mol. Cell.

[16] Rinn J.L., Chang H.Y. (2012). Genome regulation by long noncoding RNAs. Annu. Rev. Biochem..

[17] Liu X., Xiong W., Ye M., Lu T., Yuan K., Chang S. (2023). Non-coding RNAs expression in SARS-CoV-2 infection: pathogenesis, clinical significance, and therapeutic targets. Signal. Transduct. Target. Ther..

[18] Vishnubalaji R., Shaath H., Alajez N.M. (2020). Protein coding and long noncoding RNA (lncRNA) transcriptional landscape in SARS-CoV-2 infected bronchial epithelial cells highlight a role for interferon and inflammatory response. Genes (Basel).

[19] Meydan C., Madrer N., Soreq H. (2020). The neat dance of COVID-19: NEAT1, DANCR, and Co-Modulated cholinergic RNAs link to inflammation. Front. Immunol..

[20] Liao W., Xu N., Zhang H., Liao W., Wang Y., Wang S. (2022). Persistent high glucose induced EPB41L4A-AS1 inhibits glucose uptake via GCN5 mediating crotonylation and acetylation of histones and non-histones. Clin. Transl. Med..

[21] Liao M., Liao W., Xu N., Li B., Liu F., Zhang S. (2019). LncRNA EPB41L4A-AS1 regulates glycolysis and glutaminolysis by mediating nucleolar translocation of HDAC2. EBioMedicine.

[22] Zhu Y., Liu Q., Liao M., Diao L., Wu T., Liao W. (2019). Overexpression of lncRNA EPB41L4A-AS1 induces metabolic reprogramming in trophoblast cells and placenta tissue of miscarriage. Mol. Ther. Nucleic Acids.

[23] Wang Y., Liu S., Li L., Li L., Zhou X., Wan M. (2022). Peritoneal M2 macrophage-derived extracellular vesicles as natural multitarget nanotherapeutics to attenuate cytokine storms after severe infections. J. Control Release.

[24] Guo Y., Li T., Xia X., Su B., Li H., Feng Y. (2021). Different profiles of antibodies and cytokines were found between severe and moderate COVID-19 patients. Front. Immunol..

[25] Di Pietro P., Abate A.C., Izzo C., Toni A.L., Rusciano M.R., Folliero V. (2025). Plasma miR-1-3p levels predict severity in hospitalized COVID-19 patients. Br. J. Pharmacol..

[26] Kansakar U., Gambardella J., Varzideh F., Avvisato R., Jankauskas S.S., Mone P. (2022). miR-142 targets TIM-1 in human endothelial cells: potential implications for stroke, COVID-19, zika, Ebola, Dengue, and other viral infections. Int. J. Mol. Sci..

[27] Mone P., Gambardella J., Wang X., Jankauskas S.S., Matarese A., Santulli G. (2021). miR-24 targets the transmembrane glycoprotein Neuropilin-1 in human brain Microvascular endothelial cells. Noncoding RNA.

[28] Gajate-Arenas M., Sirvent-Blanco C., García-Pérez O., Domínguez-de-Barros A., Piñero J.E., Lorenzo-Morales J. (2025). miR-27a-5p, miR-21-5p, miR-1246 and miR-4508: a candidate microRNA signature in the protection and regulation of viral infection in mild COVID-19. Mol. Med..

[29] Timofeeva A.M., Nikitin A.O., Nevinsky G.A. (2024). Circulating miRNAs in the plasma of Post-COVID-19 patients with typical recovery and those with Long-COVID symptoms: regulation of immune response-associated pathways. Noncoding RNA.

[30] Xu J., Abdulsalam Khaleel R., Zaidan H.K., Faisal Mutee A., Fahmi Fawy K., Gehlot A. (2024). Discovery of common molecular signatures and drug repurposing for COVID-19/Asthma comorbidity: ACE2 and multi-partite networks. Cell Cycle.

[31] Siguemoto J.T., Motta Neri C., de Godoy Torso N., de Souza Nicoletti A., Berlofa Visacri M., Regina da Silva Correa da Ronda C. (2024). Data normalization of plasma miRNA profiling from patients with COVID-19. Sci. Rep..

[32] Wyler E., Mösbauer K., Franke V., Diag A., Gottula L.T., Arsiè R. (2021). Transcriptomic profiling of SARS-CoV-2 infected human cell lines identifies HSP90 as target for COVID-19 therapy. iScience.

[33] Daamen A.R., Bachali P., Owen K.A., Kingsmore K.M., Hubbard E.L., Labonte A.C. (2021). Comprehensive transcriptomic analysis of COVID-19 blood, lung, and airway. Sci. Rep..

[34] Daniloski Z., Jordan T.X., Wessels H.H., Hoagland D.A., Kasela S., Legut M. (2021). Identification of required host factors for SARS-CoV-2 infection in human cells. Cell.

[35] Hoagland D.A., Clarke D.J.B., Møller R., Han Y., Yang L., Wojciechowicz M.L. (2020). Modulating the transcriptional landscape of SARS-CoV-2 as an effective method for developing antiviral compounds. bioRxiv.

[36] Sims A.C., Tilton S.C., Menachery V.D., Gralinski L.E., Schäfer A., Matzke M.M. (2013). Release of severe acute respiratory syndrome coronavirus nuclear import block enhances host transcription in human lung cells. J. Virol..

[37] Josset L., Menachery V.D., Gralinski L.E., Agnihothram S., Sova P., Carter V.S. (2013). Cell host response to infection with novel human coronavirus EMC predicts potential antivirals and important differences with SARS coronavirus. mBio.

[38] Menachery V.D., Eisfeld A.J., Schäfer A., Josset L., Sims A.C., Proll S. (2014). Pathogenic influenza viruses and coronaviruses utilize similar and contrasting approaches to control interferon-stimulated gene responses. mBio.

[39] Lao B.B., Grishagin I., Mesallati H., Brewer T.F., Olenyuk B.Z., Arora P.S. (2014). In vivo modulation of hypoxia-inducible signaling by topographical helix mimetics. Proc. Natl. Acad. Sci. U. S. A..

[40] Jain R., Ramaswamy S., Harilal D., Uddin M., Loney T., Nowotny N. (2021). Host transcriptomic profiling of COVID-19 patients with mild, moderate, and severe clinical outcomes. Comput. Struct. Biotechnol. J..

[41] Deguchi S., Kosugi K., Hashimoto R., Sakamoto A., Yamamoto M., Krol R.P. (2023). Elucidation of the liver pathophysiology of COVID-19 patients using liver-on-a-chips. PNAS Nexus.

[42] Li C., Tang Z., Zhang W., Ye Z., Liu F. (2021). GEPIA2021: integrating multiple deconvolution-based analysis into GEPIA. Nucleic Acids Res..

