# Supplementary material for: Long non-coding RNA enhances SARS-CoV-2-mediated apoptosis through epigenetic repression of angiotensin-converting enzyme 2
Source: J Biol Chem. 2025 Oct 13;301(12):110812. doi: 10.1016/j.jbc.2025.110812 (PMC12639495; doi:10.1016/j.jbc.2025.110812)
Supplement: Supporting Information 1 [file mmc1.docx]

**Supplementary materials**

1. **Supplementary Figures**

**
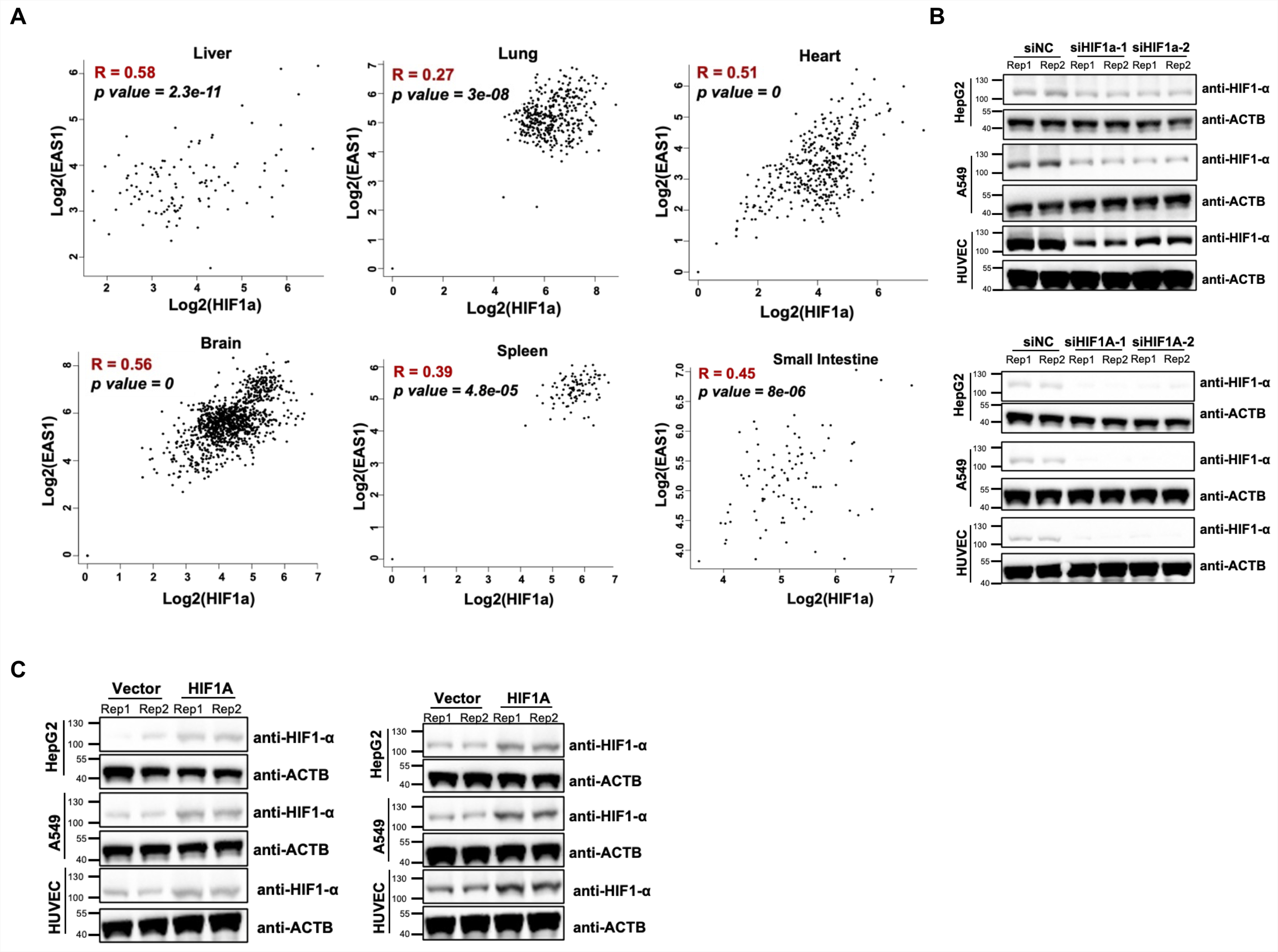
**

**Figure S1. Correlation analysis and additional validation for HIF-1α-mediated EAS1 induction.** (A) Scatter plots showing the Pearson correlation between HIF1A and EAS1 expression using RNAseq data of different tissues from GTEx database, each dot represents a tissue sample; (B-C) Western blot analysis from two additional independent biological replicates corresponding to Figure 2F, validating HIF-1α knockdown (KD) and overexpression (OE) efficiency.


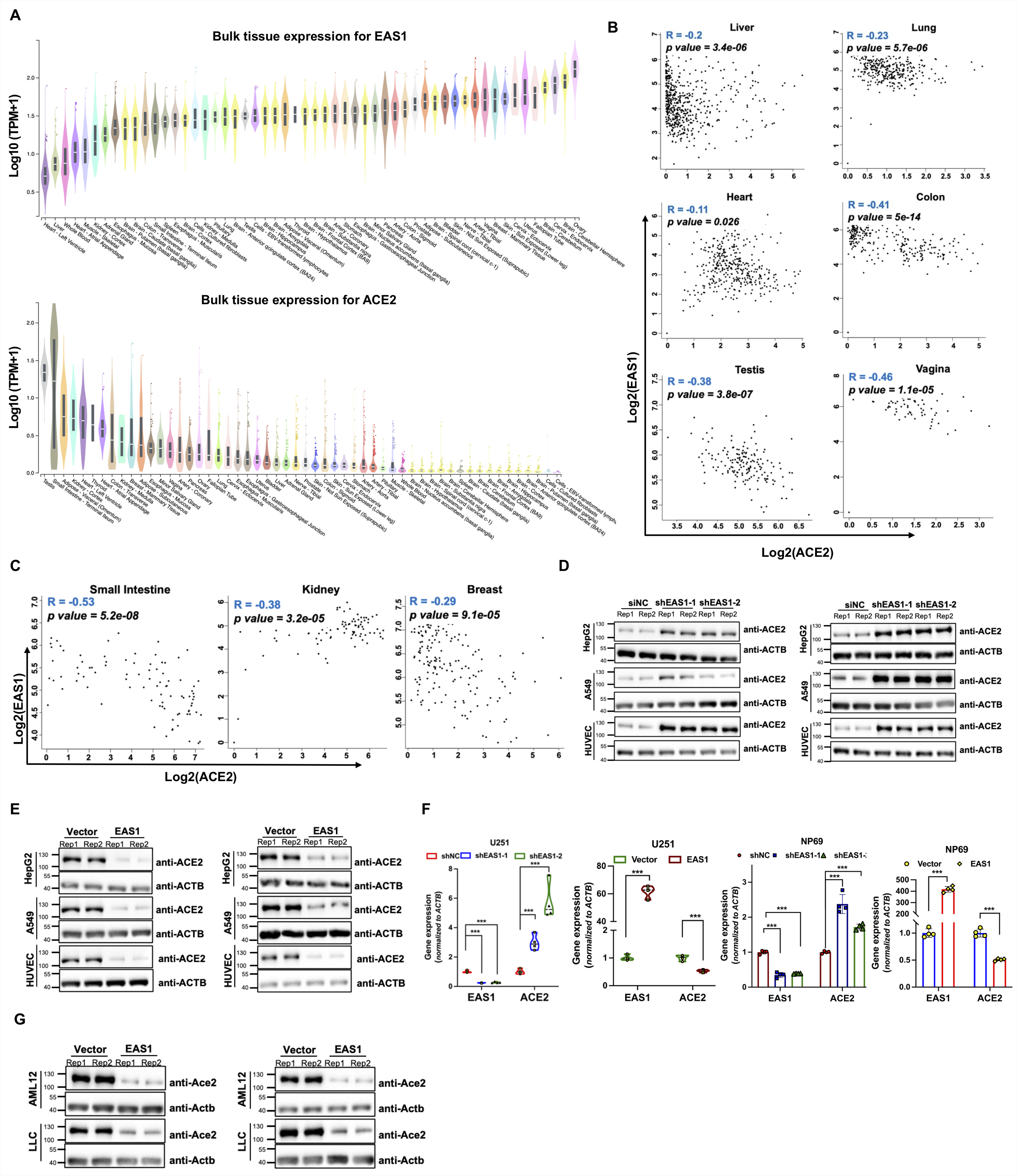


**Figure S2. EAS1 is negatively correlated with ACE2 and additional supporting data.** (A) Bar graph depicting the relative expression of EAS1 and ACE2 across various human tissues. Data were from GTEx database; (B-C) Scatter plots showing the negative Pearson correlation between ACE2 and EAS1 expression using RNAseq data of different tissues from GTEx database; (D-E) Two additional independent biological replicates of western blot corresponding to Figure 3G; (F) qPCR quantification of ACE2 mRNA levels in additional cell lines after EAS1 KD or OE (n = 3-4, each in triplicate). Data are mean ± SD. Statistical significance was determined by a two-tailed paired Student's t-test, ***p<0.001, **p<0.01, *p<0.05. (G) Western blot analysis from two additional independent biological replicates corresponding to Figure 3M.


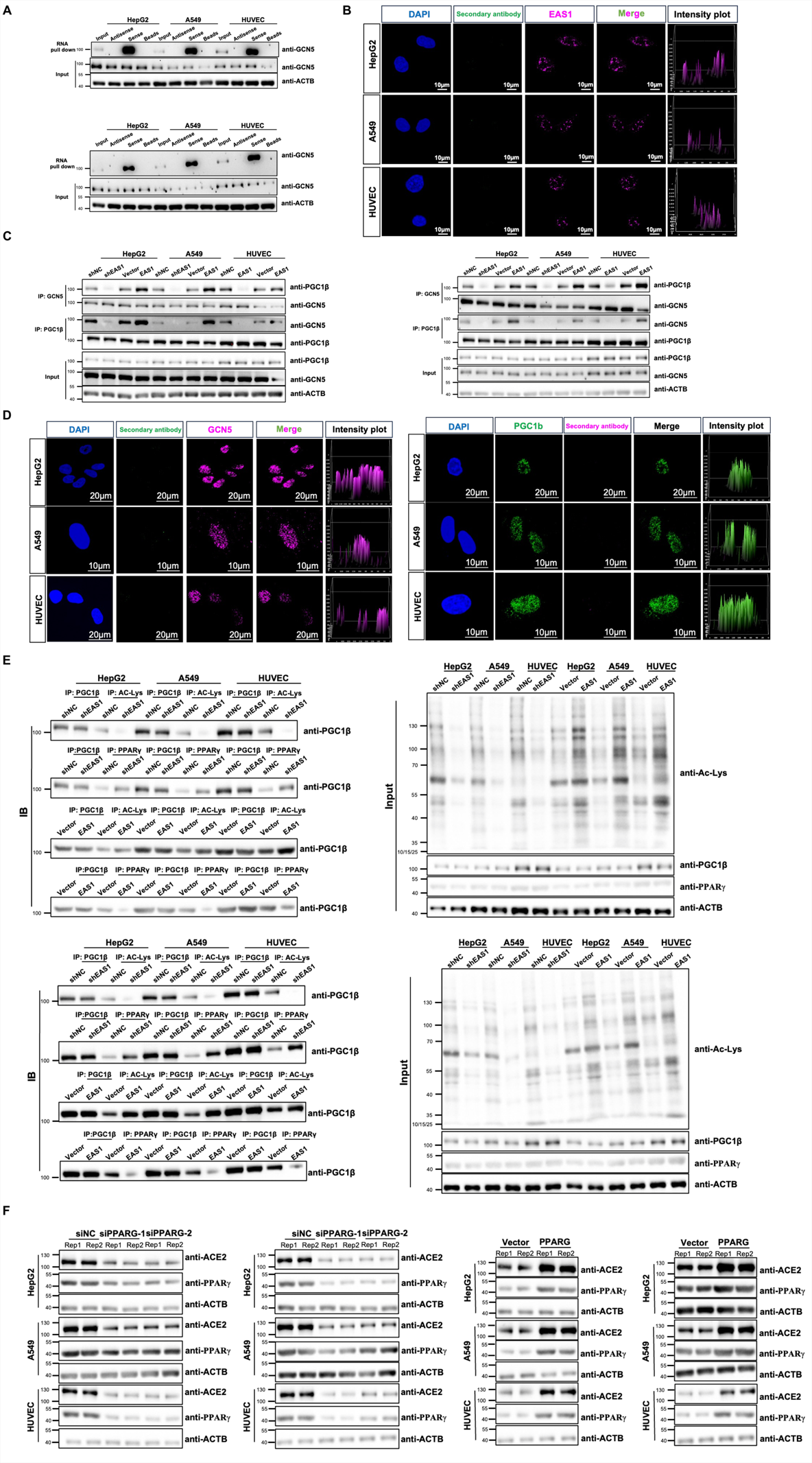


**Figure S3. Additional controls and validation for the EAS1-GCN5-PGC1β-PPARγ axis.** (A) Two additional independent biological replicates of western blot corresponding to Figure 4C; (B) Negative control for RNA FISH/IF co-localization assay. Cells were processed identically to those in Figure 4E but incubated with a fluorophore-conjugated secondary antibody only (no primary anti-GCN5 antibody), scale bars = 10µm; (C) Two additional independent biological replicates of western blot related to Figure 4F; (D) Negative controls for IF co-localization assays. Cells were incubated with secondary antibodies only (no primary antibodies against GCN5 or PGC1β), scale bars were shown in the picture. (E) Two additional independent biological replicates of western blot related to Figure 4I; (F) Two additional independent biological replicates of western blot related to Figure 4J.


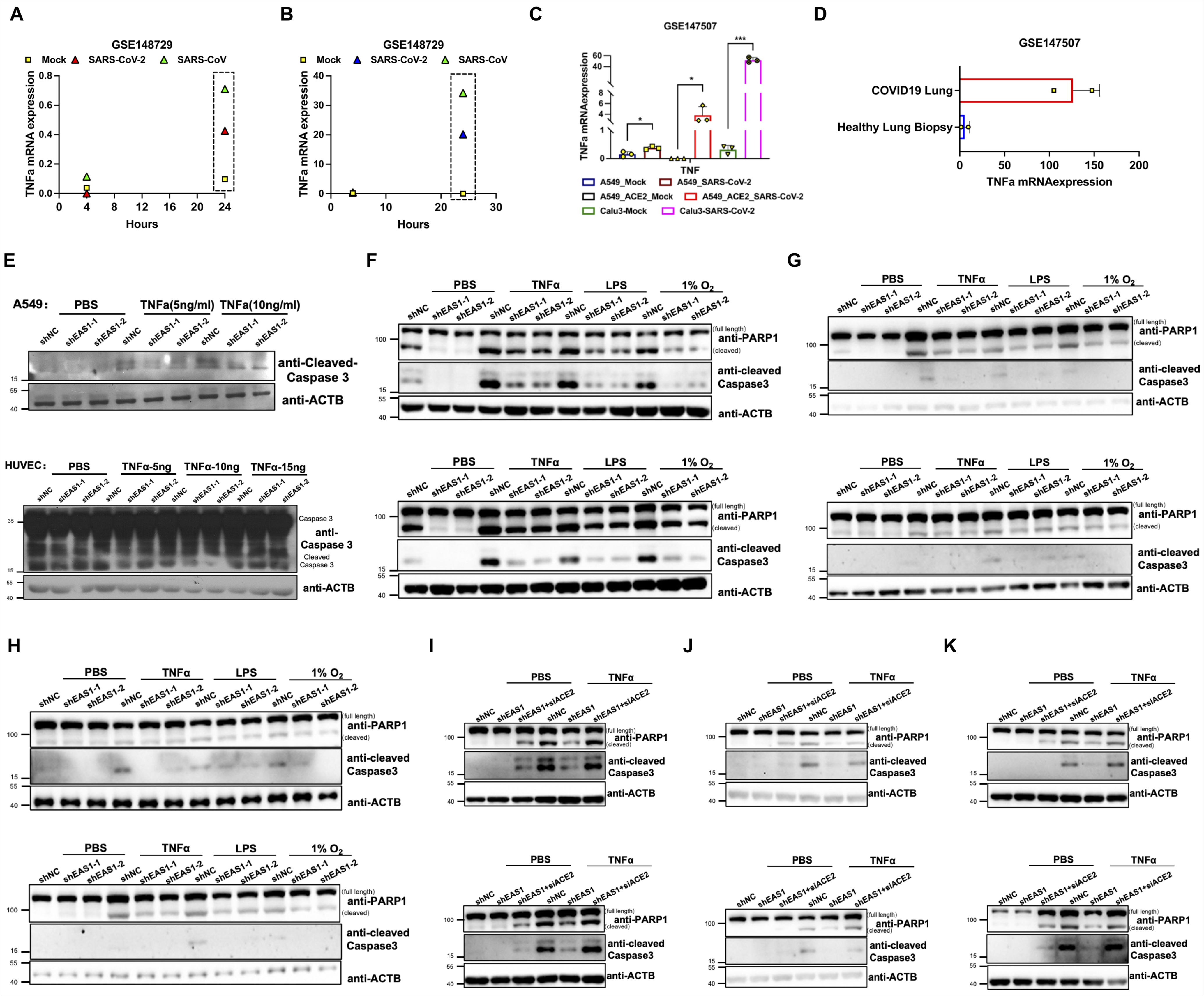


**Figure S4. Additional apoptosis data that inhibition of EAS1 attenuates SARS-CoV-2 infection-related apoptosis**. (A-B) RNA-seq analysis of TNFα expression in Caco-2 (A) and Calu-3 (B) cells infected with SARS-CoV-2 or SARS-CoV at the indicated time points (n = 2); (C) TNFα mRNA levels in A549, ACE2-A549, and Calu-3 cells 24 hours post-infection (hpi) with SARS-CoV-2 (n = 3). Statistical significance was determined by a two-tailed paired Student's t-test, ***p<0.001, **p<0.01, *p<0.05; (D) RNA-seq analysis of TNFα levels in the lungs of COVID-19 deceased patients (n = 2) and healthy individuals (n = 2); (E) Western blot analysis of apoptosis markers (cleaved Caspase-3) in EAS1 KD and control cells treated with the indicated concentrations of TNFα for 24 hours to establish the optimal dose (Representative data from one experiment); (F-K) Western blot analysis from two additional independent biological replicates corresponding to Figure 5C (F), 5E (G), 5F (H), 5L (I), 5M (J), and 5O (K).

1. **Supplementary Tables**

**Table S1: Reagents**

| **Reagent** | **Source** | **Identifier** |
| --- | --- | --- |
| Lipofectamine3000 | Invitrogen | L3000015 |
| Puromycin | Gibco | A1113803 |
| Lipopolysaccharides | MedChemExpress | HY-P70426 |
| TNFα | MedChemExpress | HY-D1056 |
| Polybrene | MedChemExpress | HY-112735 |
| ITS | Sigma-Aldrich | I3146 |
| Dexamethasone | Sigma-Aldrich | 50-02-2 |
| Collagenase IV | Sigma-Aldrich | C5138 |
| EGTA | Sigma-Aldrich | E4378 |
| Collagenase I | Sigma-Aldrich | C0130 |
| Rat Tail Collagen, Type I | Corning | 354236 |
| Collagenase II | Sigma-Aldrich | C6885 |
| Penicillin-Streptomycin | Gibco | 15140122 |

**Table S2: Sequences of the small interfering RNAs**

| **Name** | **Sense** | **Antisense** |
| --- | --- | --- |
| siHIF1A-1 | CGAUGGAAGCACUAGACAAAG | UUGUCUAGUGCUUCCAUCGGA |
| siHIF1A-2 | GAUGGAAGCACUAGACAAAGU | UUUGUCUAGUGCUUCCAUCGG |
| siEAS1-1 | GGAUGUCCUUGGUGAGGAUTT | ATCCTCACCAAGGACATCCTT |
| siEAS1-2 | GCCUGUCCUUUCUUCCUUUTT | AAAGGAAGAAAGGACAGGCTT |
| siPPARγ | CAUUCCAUUCACAAGAACAGA | UGUUCUUGUGAAUGGAAUGUC |
| siACE2 | CCAUCUACAGUACUGGAAATT | UUUCCAGUACUGUAGAUGGTT |
| siNC | UUCUCCGAACGUGUCACGUTT | ACGUGACACGUUCGGAGAATT |

**Table S3: Sequences of the primers used to detect genes expression by qPCR**

| **Name** | **Forward** | **Reverse** |
| --- | --- | --- |
| ACTB | TGACGTGGACATCCGCAAAG | CTGGAAGGTGGACAGCGAGG |
| EAS1 | CCTGGTTTTATTTTCGTCA | ATCCATCTTCCACCTGTAG |
| ACE2 | ACAGTCCACACTTGCCCAAAT | TGAGAGCACTGAAGACCCATT |
| HIF1A | ACAGTATTCCAGCAGACTCAA | CCTACTGCTTGAAAAAGTGAA |
| ChIP-HIF1A | GGCGTAAATTAAACGCTTTG | AGAACTGGCTAAGAGAAACC |
| PPARγ | CGAAGACATTCCATTCACAA | CCACAGACACGACATTCA |
| ChIP-PPARγ | TACCCTCATCTCACTTTCA | TGGACTCTTACTTCCCTTG |

**Table S4: Characterization of the primary antibodies used in the study**

| **Target** | **Type** | **Supplier** |
| --- | --- | --- |
| ACTB | Monoclonal | Proteintech |
| HIF1α | Polyclonal | Proteintech |
| ACE2 | Polyclonal | Proteintech |
| GCN5 | Monoclonal | Proteintech |
| PGC1β | Polyclonal | Proteintech |
| Acetylated-Lysine | Polyclonal | Cell Signaling Technology |
| PPARγ | Polyclonal | Proteintech |
| Caspase3 | Monoclonal | Abcam |
| Cleaved Caspase3 | Monoclonal | Abcam |
| PARP1 | Polyclonal | Proteintech |
