# Supplementary material for: Long non-coding RNA enhances SARS-CoV-2-mediated apoptosis through epigenetic repression of angiotensin-converting enzyme 2
Source: J Biol Chem. 2025 Oct 13;301(12):110812. doi: 10.1016/j.jbc.2025.110812 (PMC12639495; doi:10.1016/j.jbc.2025.110812)
Supplement: Supporting Information 2 [file mmc2.pptx]

## Slide 1
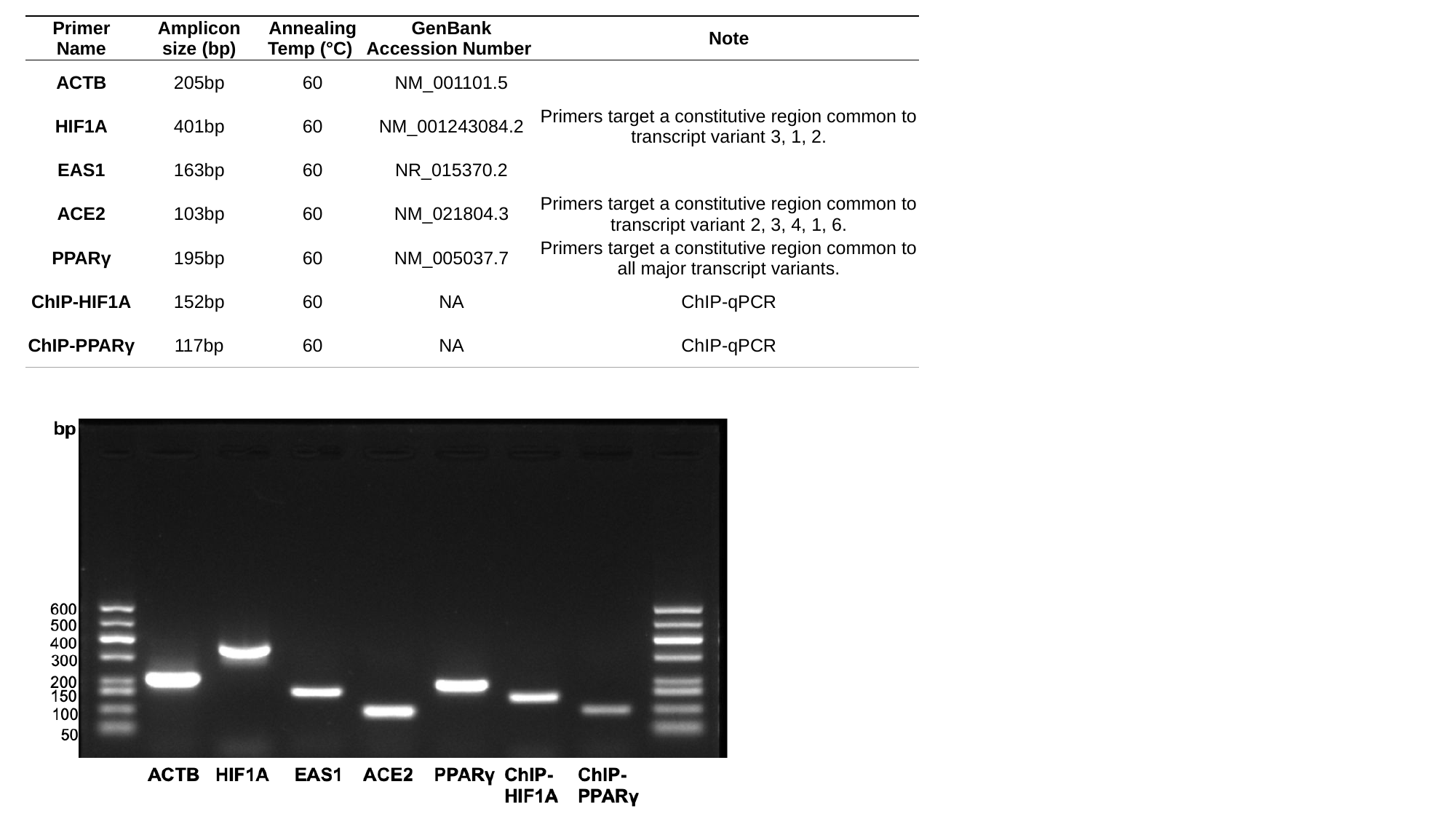

| Primer Name | Amplicon size (bp) | Annealing Temp (°C) | GenBank Accession Number | Note |
| --- | --- | --- | --- | --- |
| ACTB | 205bp | 60 | NM\_001101.5 | |
| HIF1A | 401bp | 60 | NM\_001243084.2 | Primers target a constitutive region common to transcript variant 3, 1, 2. |
| EAS1 | 163bp | 60 | NR\_015370.2 | |
| ACE2 | 103bp | 60 | NM\_021804.3 | Primers target a constitutive region common to transcript variant 2, 3, 4, 1, 6. |
| PPARγ | 195bp | 60 | NM\_005037.7 | Primers target a constitutive region common to all major transcript variants. |
| ChIP-HIF1A | 152bp | 60 | NA | ChIP-qPCR |
| ChIP-PPARγ | 117bp | 60 | NA | ChIP-qPCR |

## Slide 2
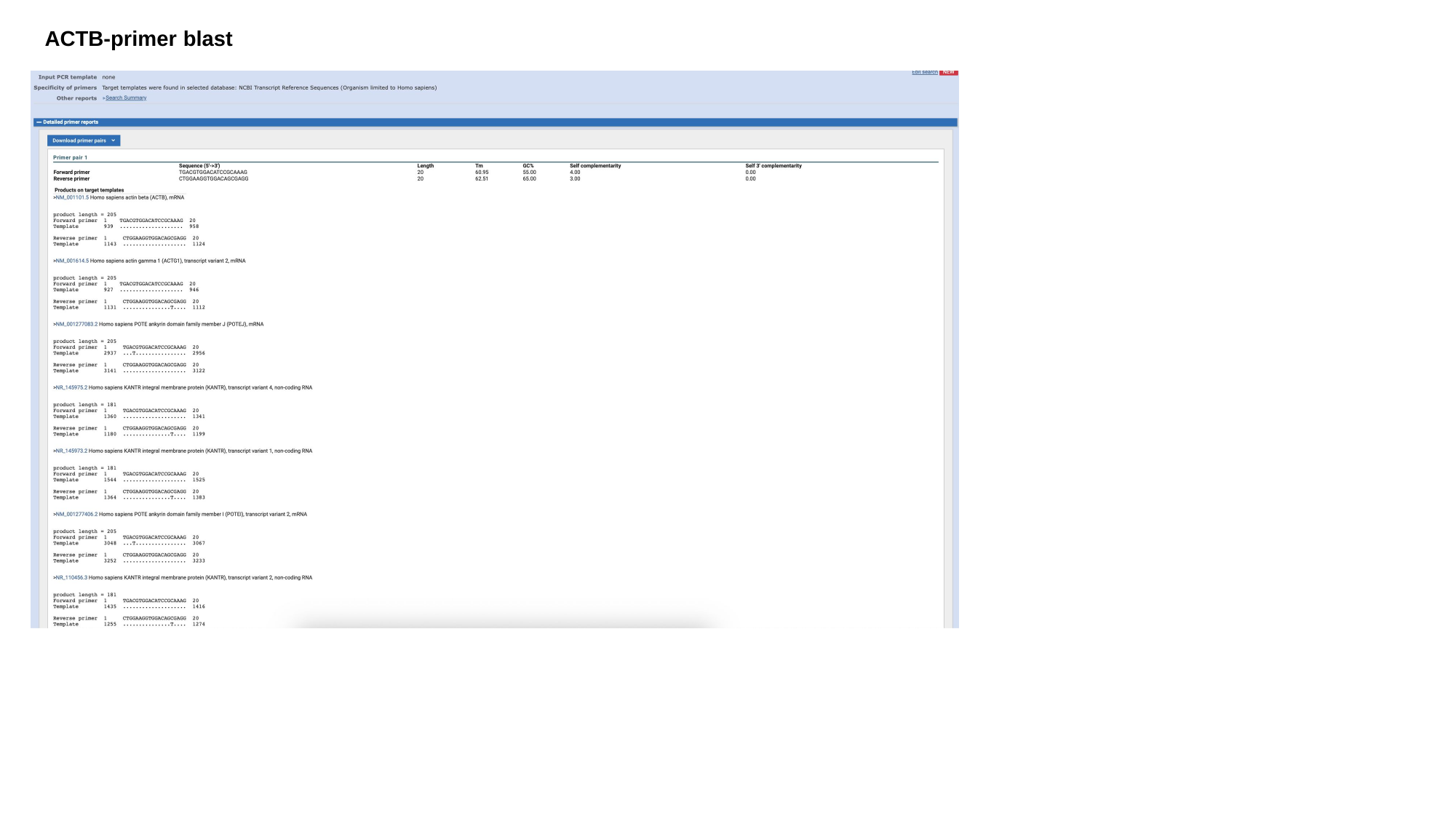

ACTB-primer blast

## Slide 3
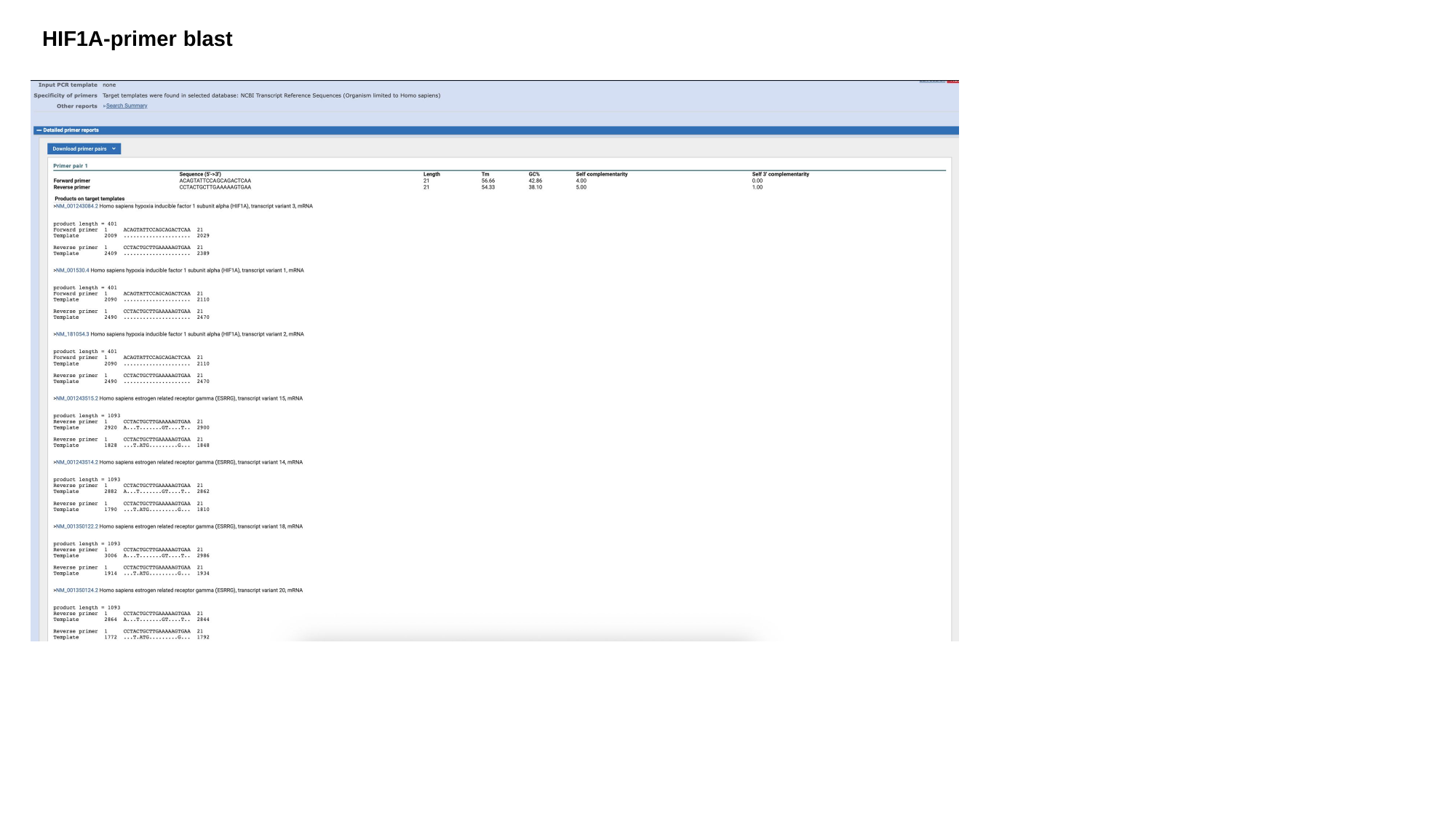

HIF1A-primer blast

## Slide 4
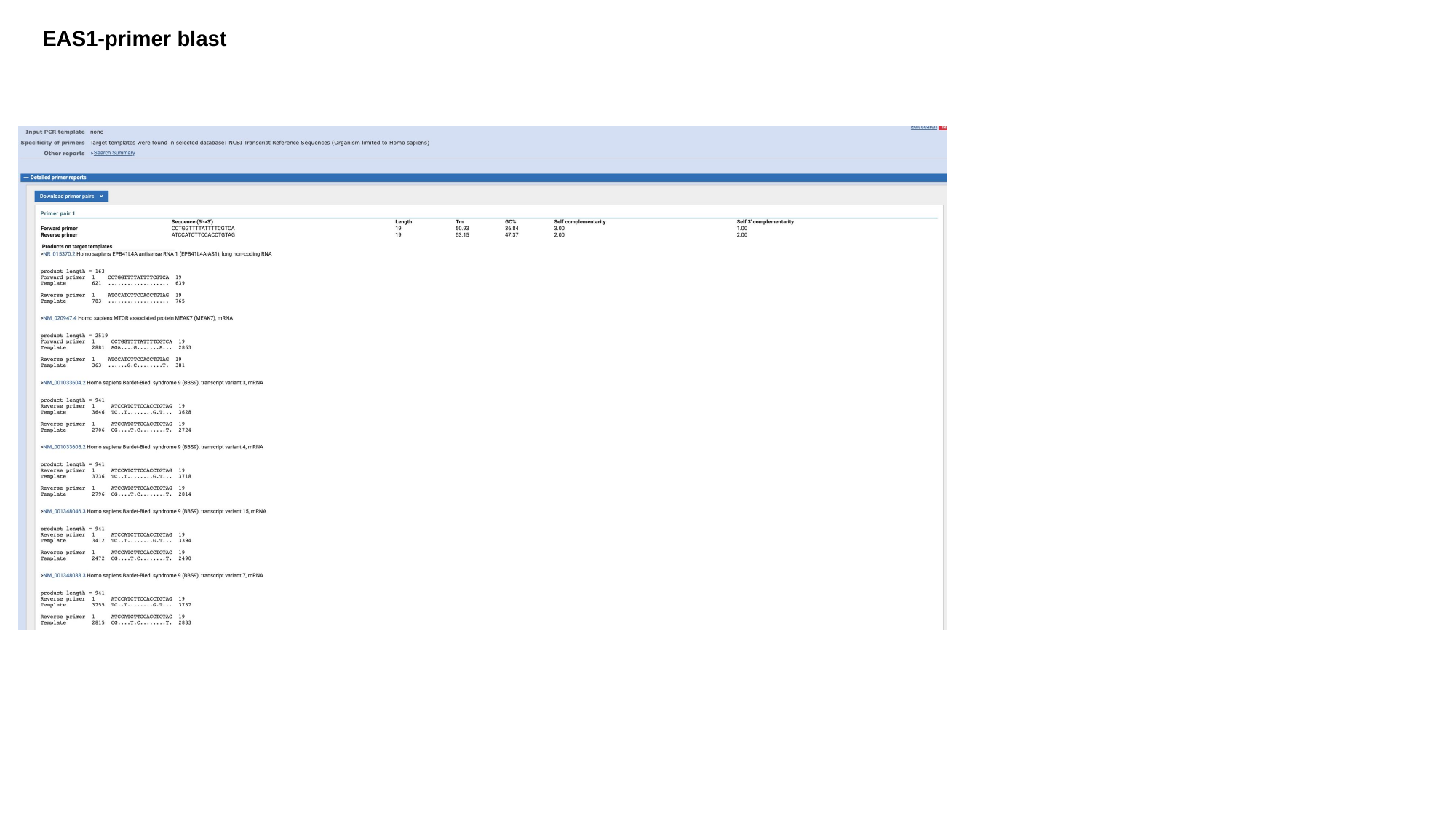

EAS1-primer blast

## Slide 5
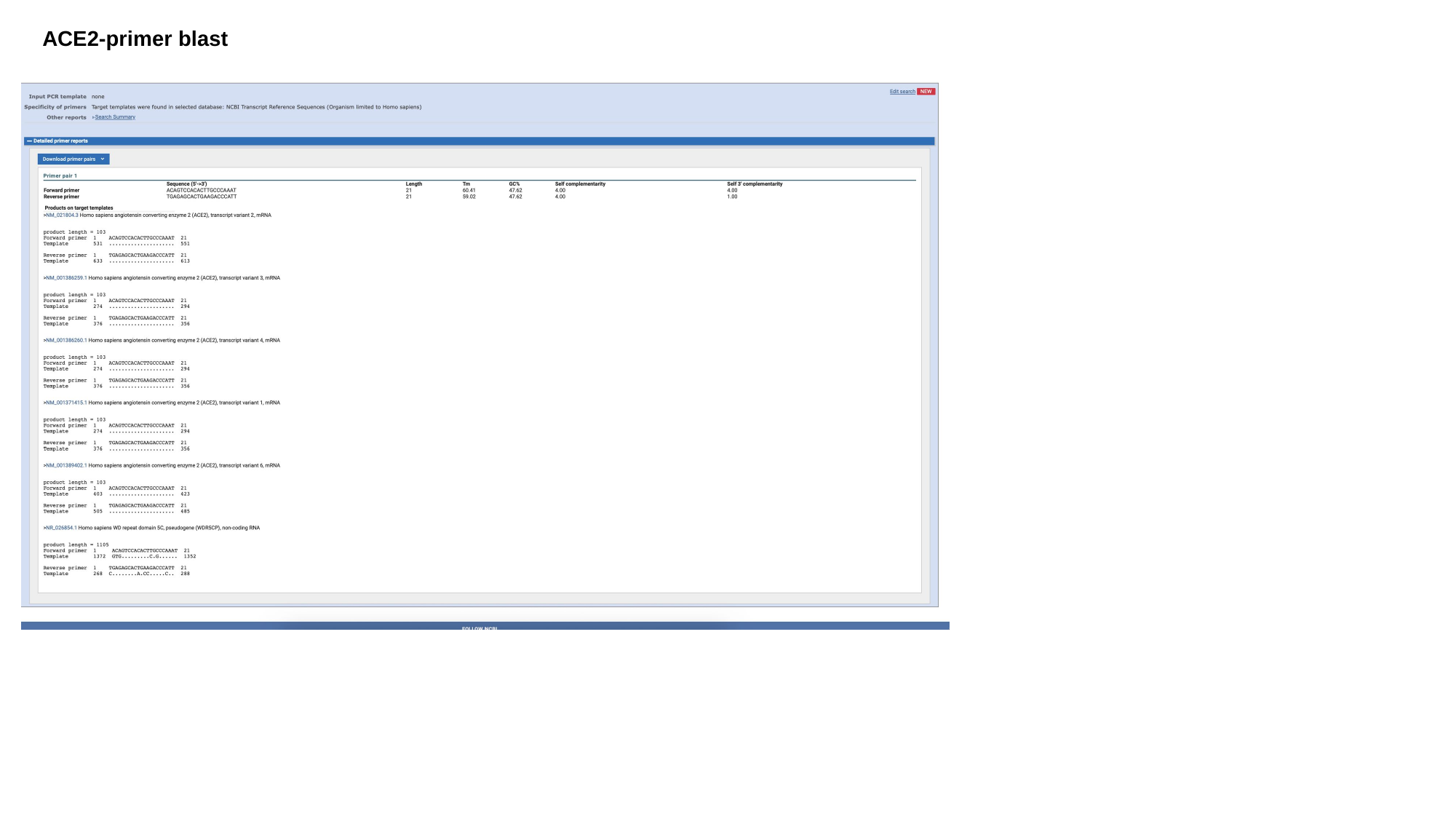

ACE2-primer blast

## Slide 6
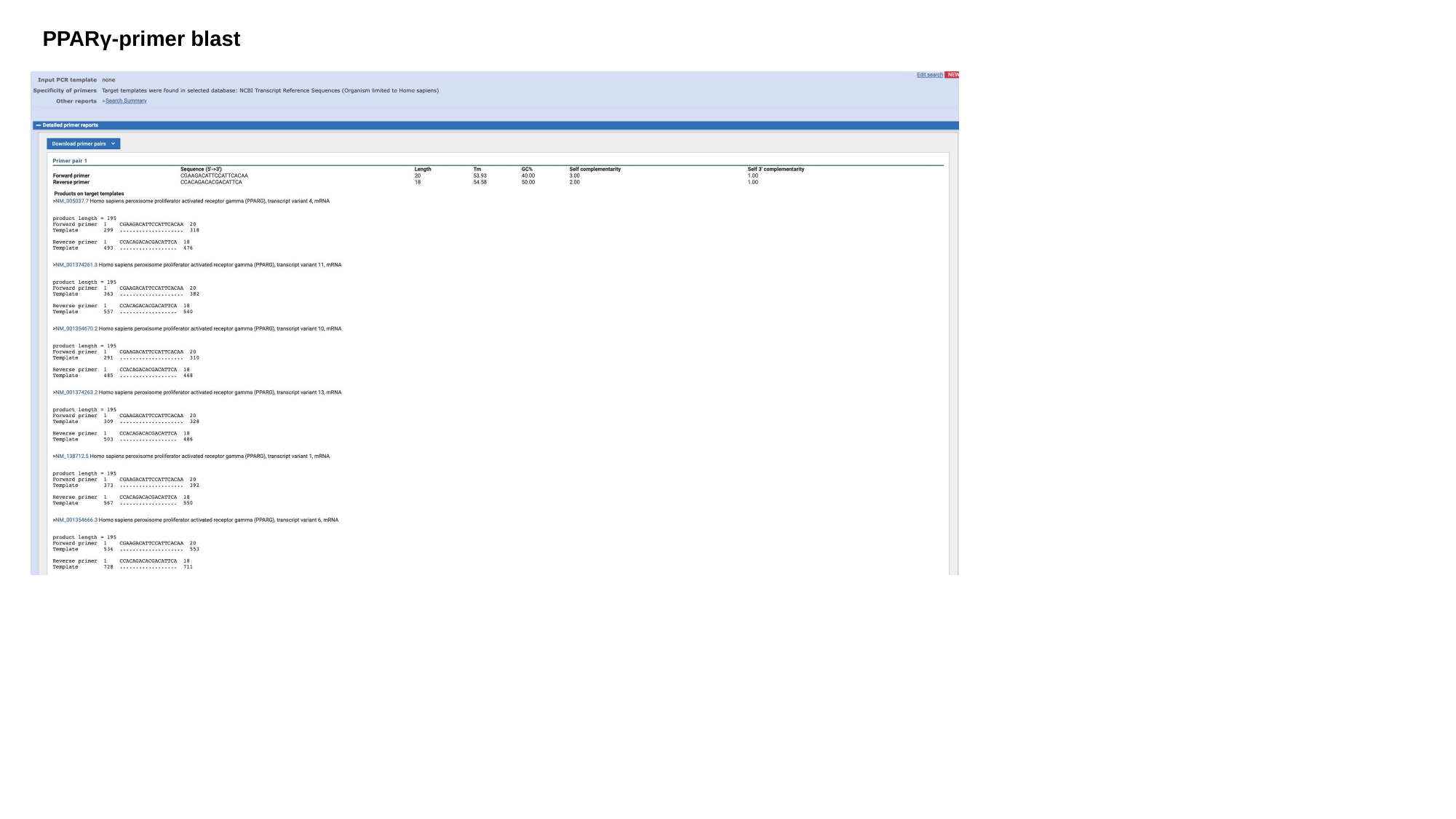

PPARγ-primer blast

## Slide 7
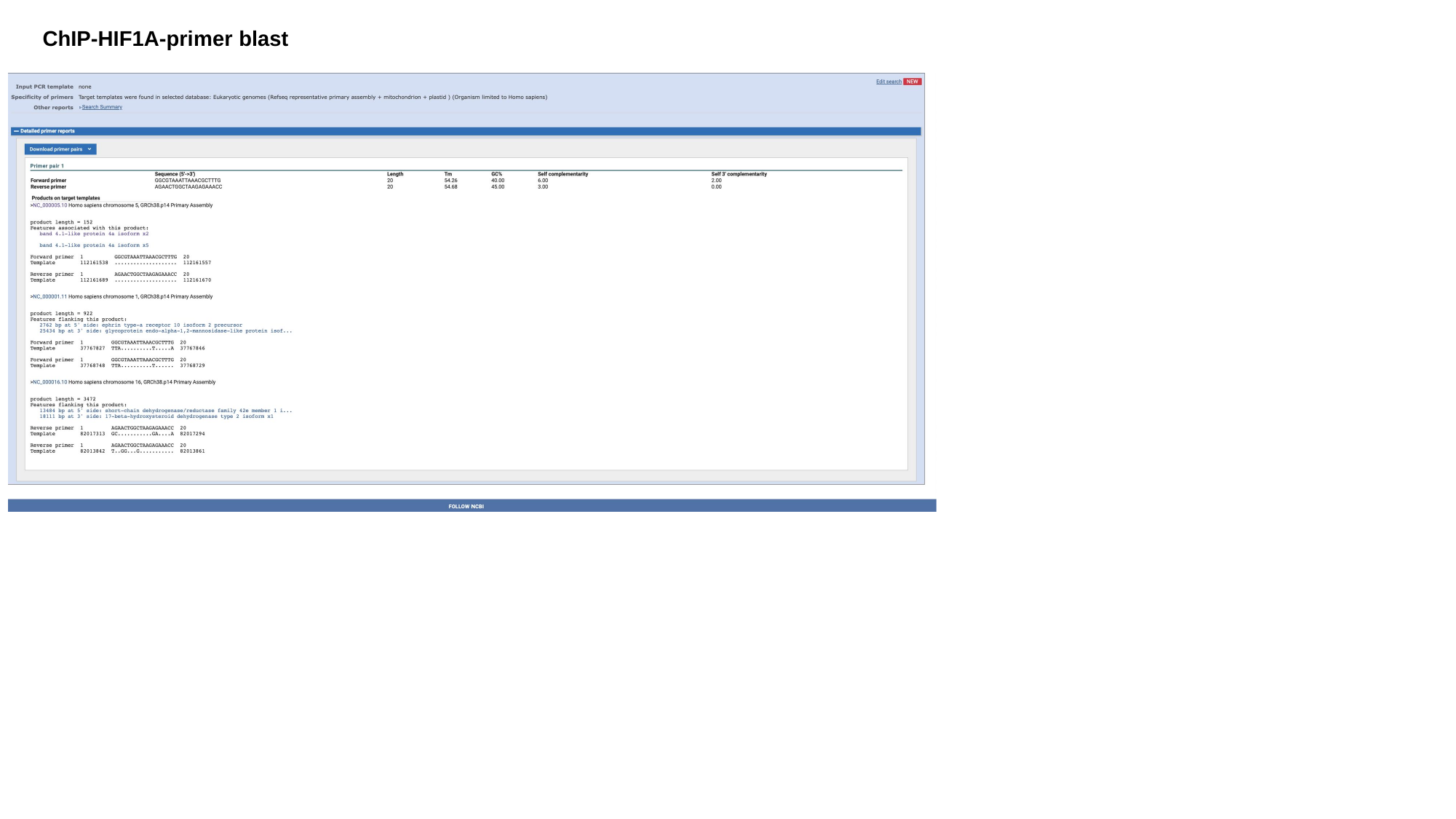

ChIP-HIF1A-primer blast

## Slide 8
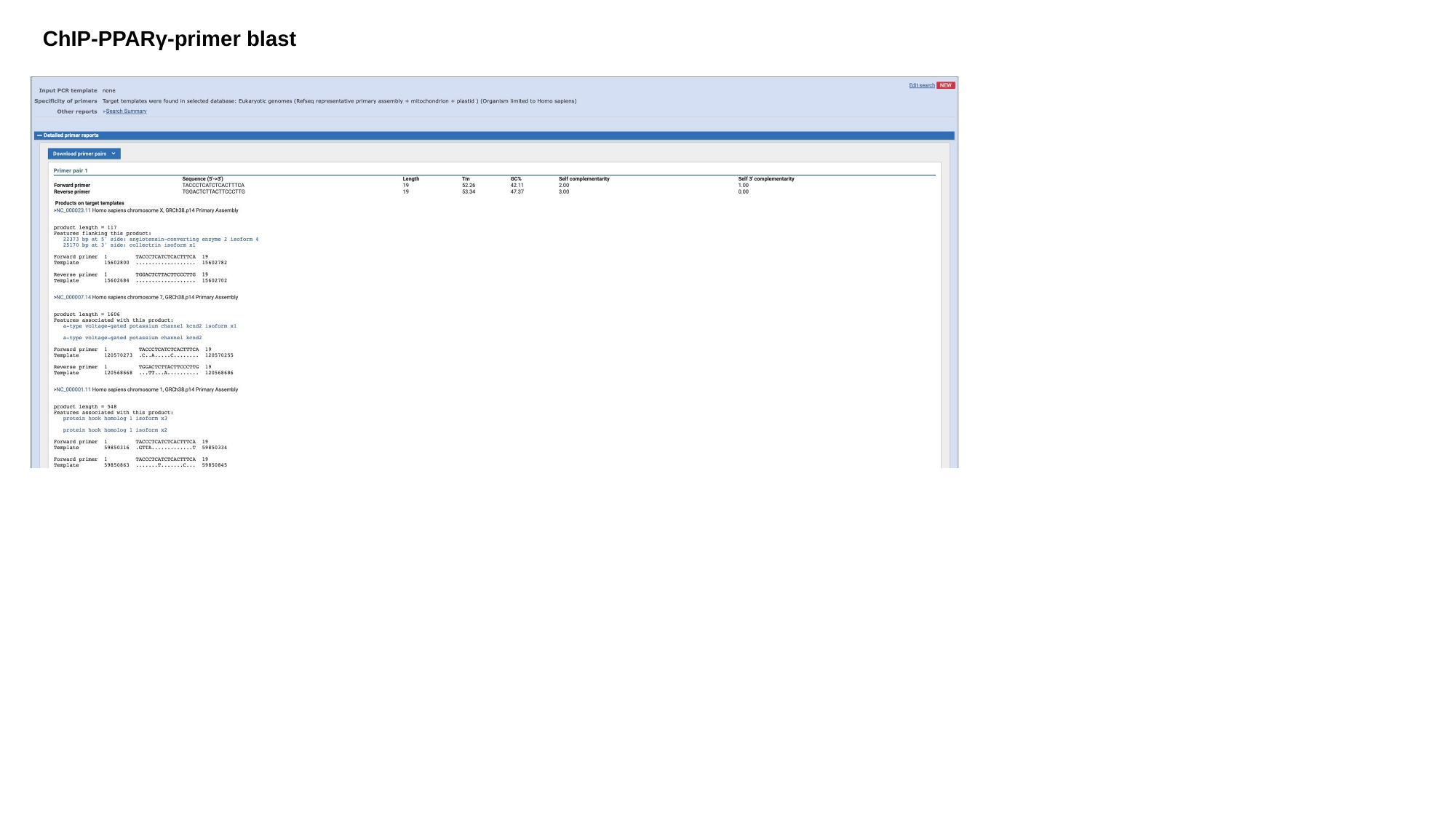

ChIP-PPARγ-primer blast

## Slide 9
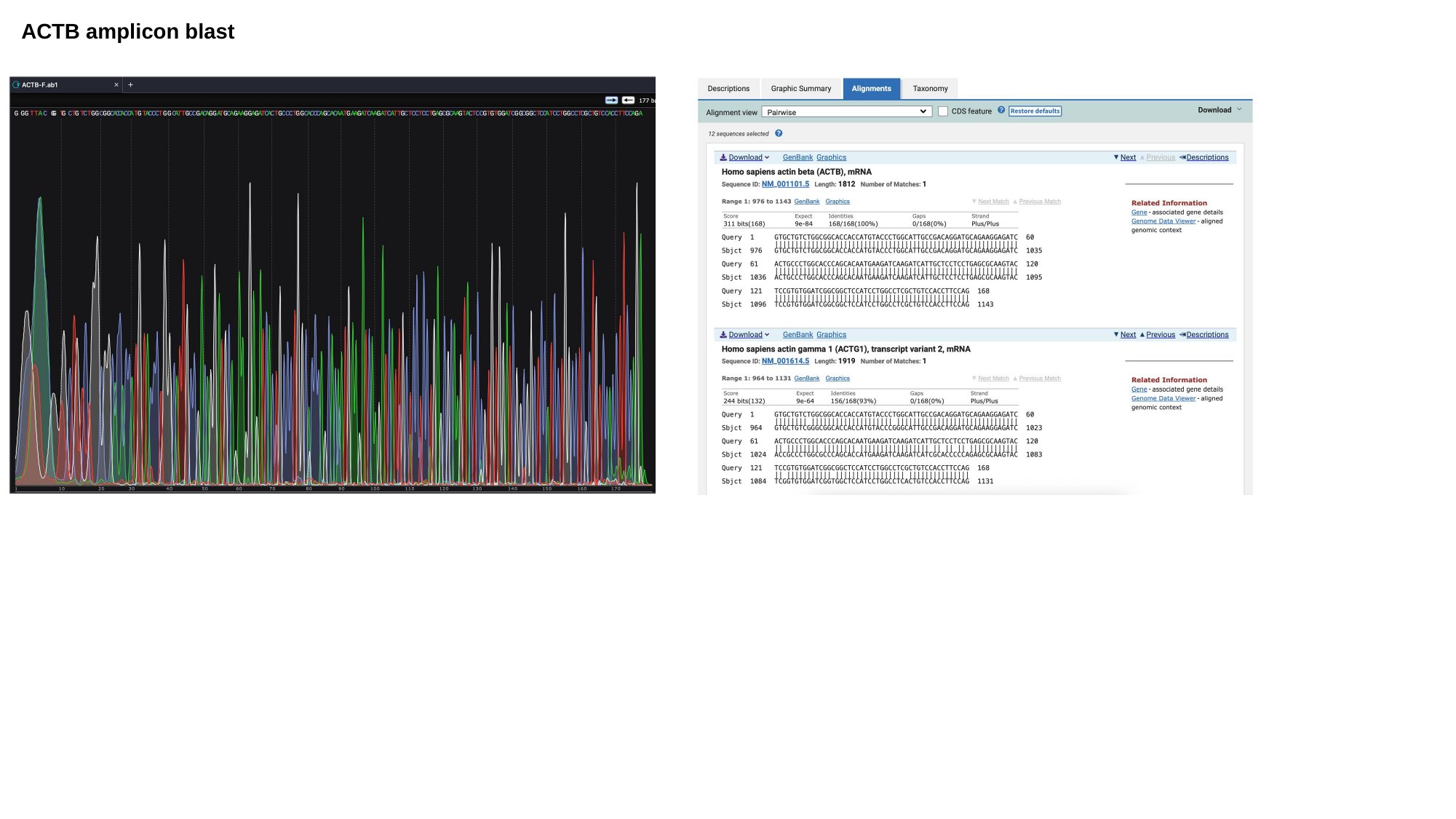

ACTB amplicon blast

## Slide 10
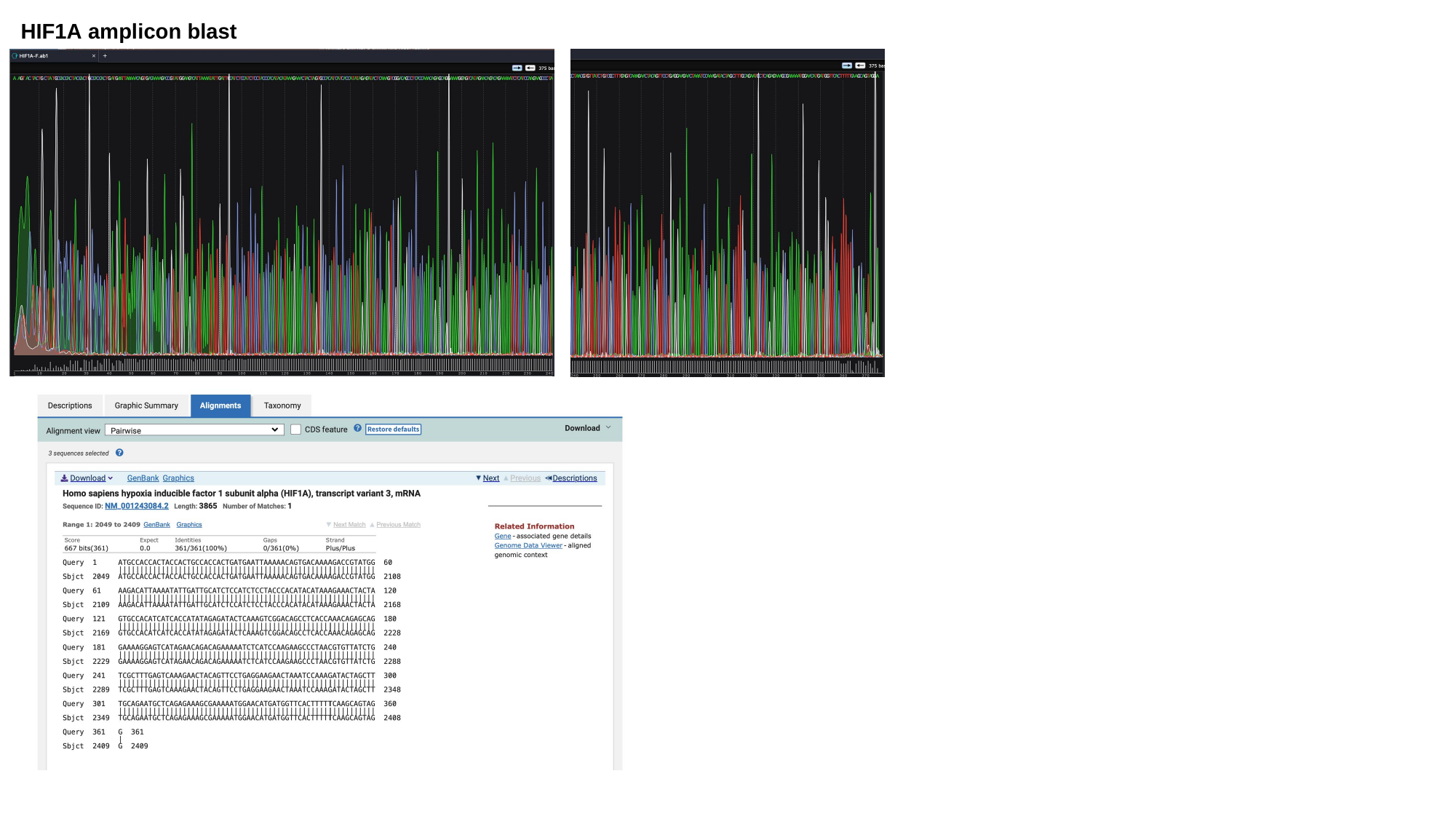

HIF1A amplicon blast

## Slide 11
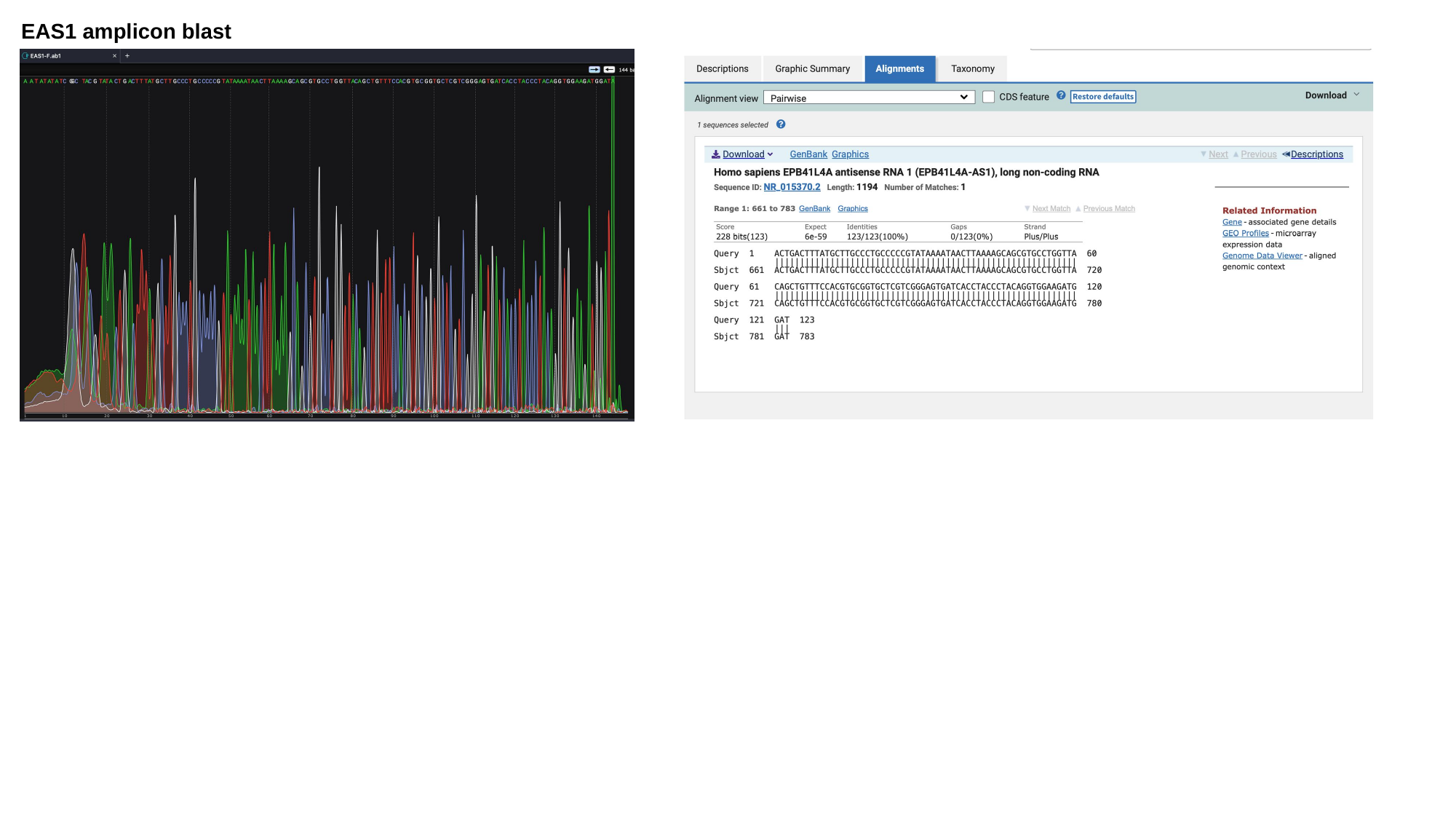

EAS1 amplicon blast

## Slide 12
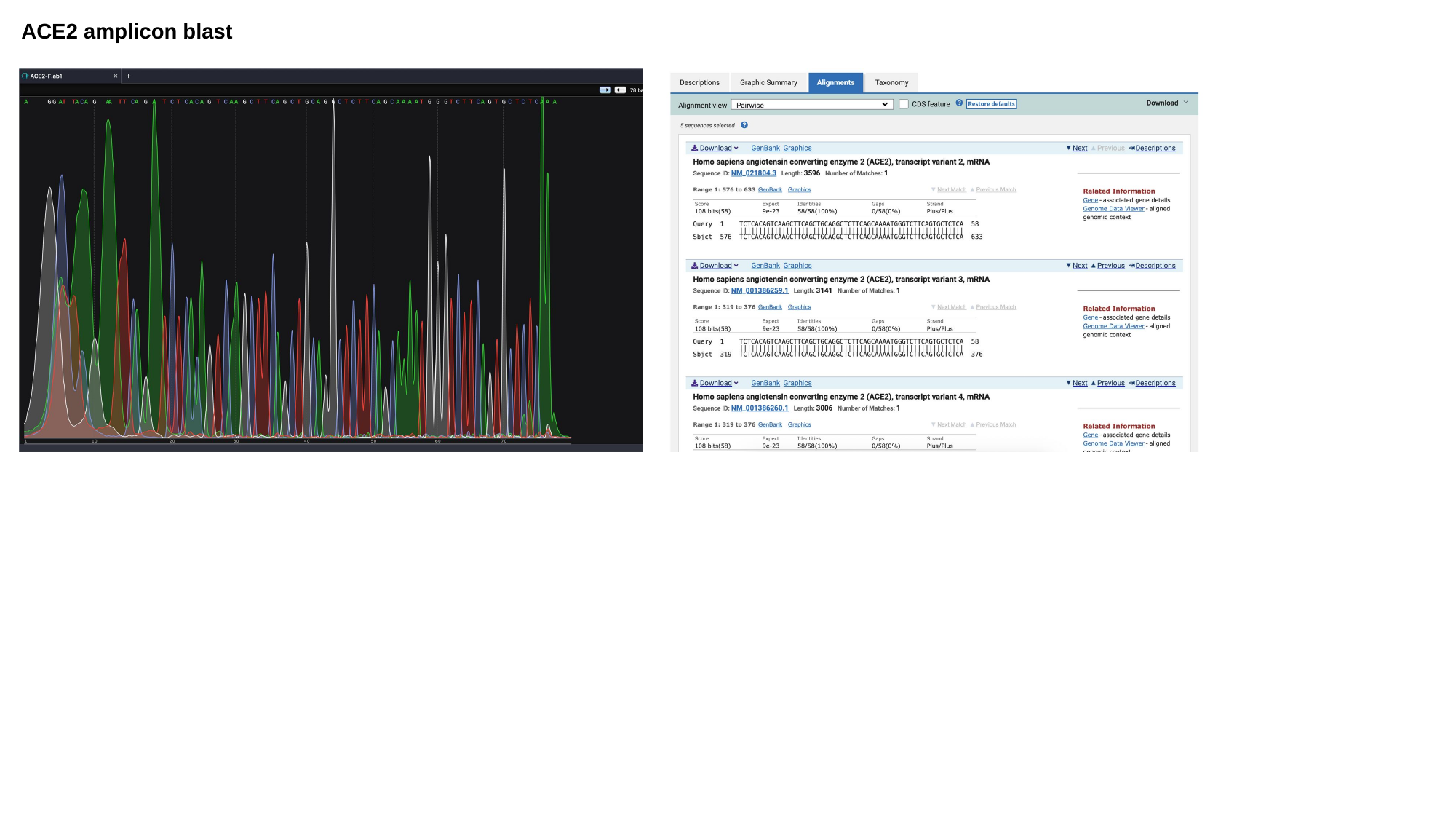

ACE2 amplicon blast

## Slide 13
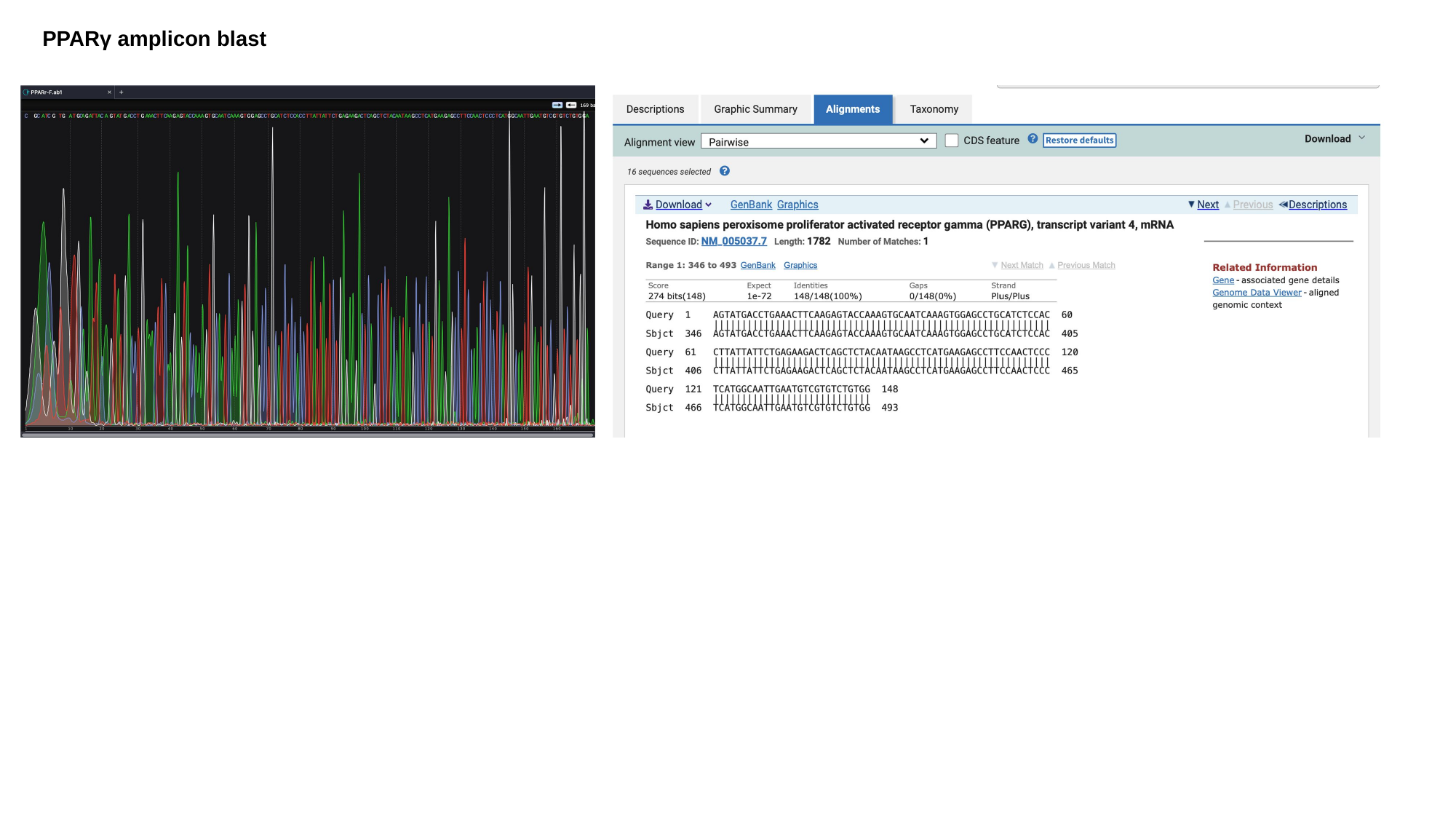

PPARγ amplicon blast

## Slide 14
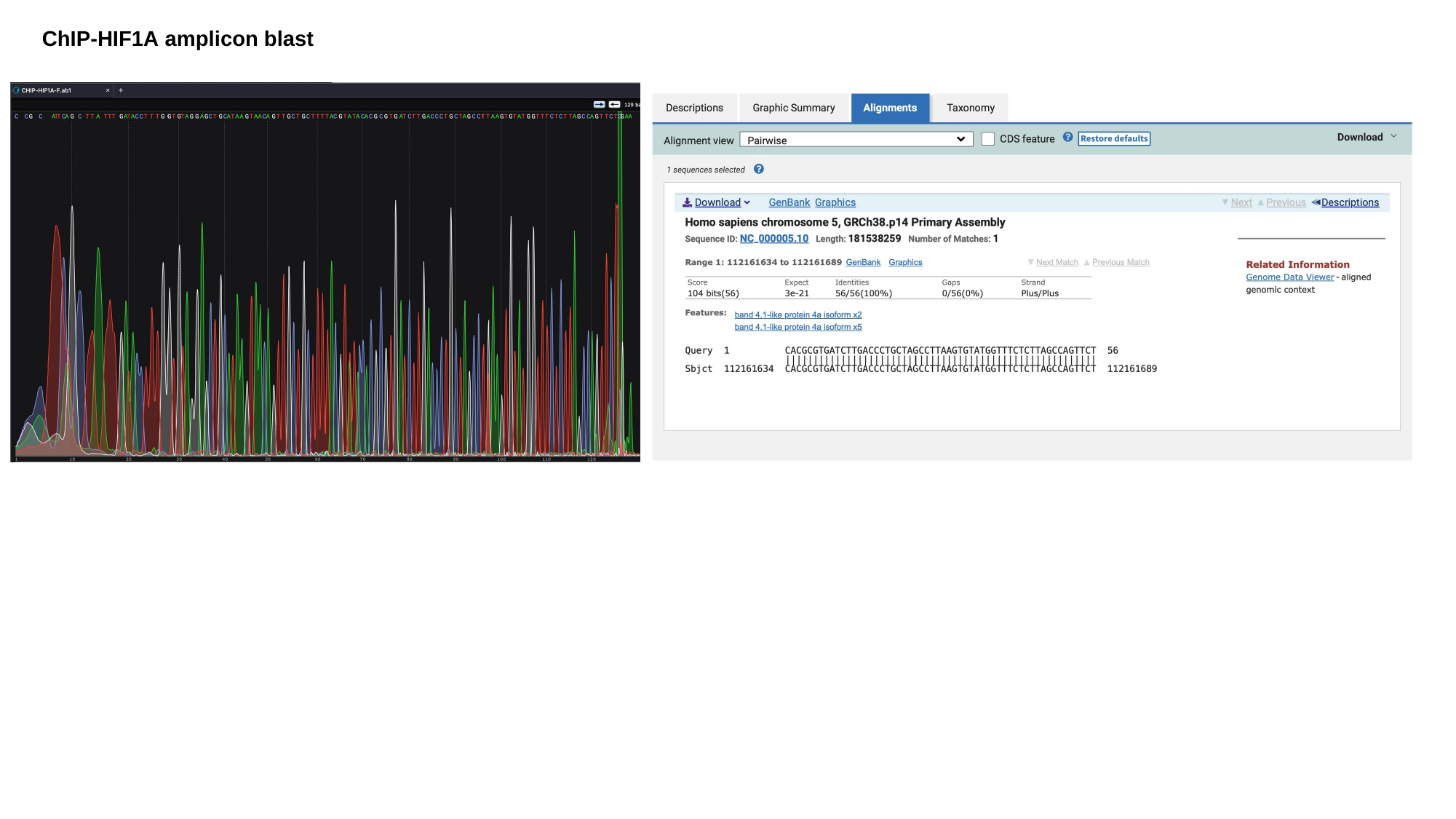

ChIP-HIF1A amplicon blast

## Slide 15
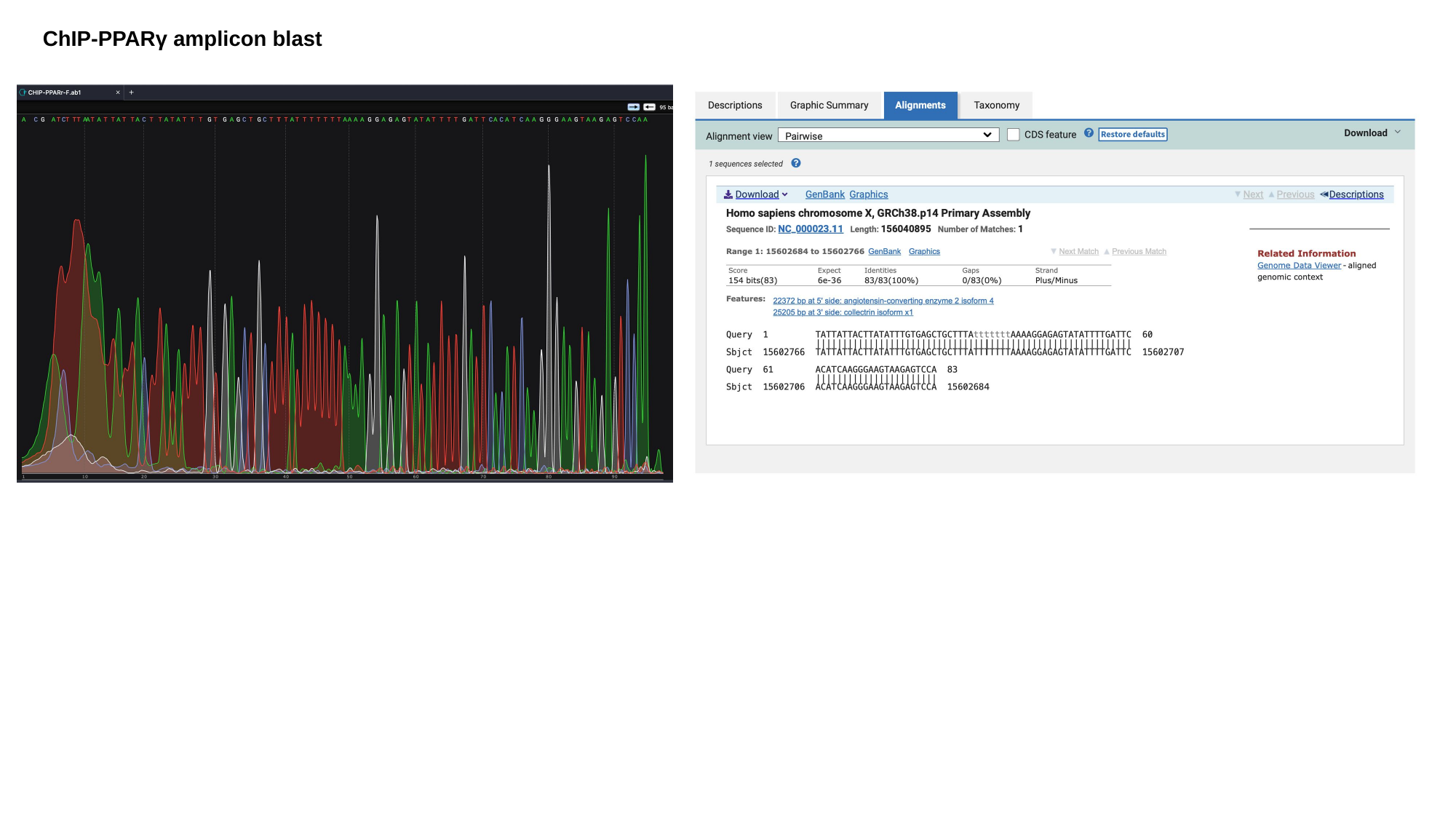

ChIP-PPARγ amplicon blast
