# Supplementary figures and images for: Long non-coding RNA enhances SARS-CoV-2-mediated apoptosis through epigenetic repression of angiotensin-converting enzyme 2
Source: J Biol Chem. 2025 Oct 13;301(12):110812. doi: 10.1016/j.jbc.2025.110812 (PMC12639495; doi:10.1016/j.jbc.2025.110812)

## Slide 1
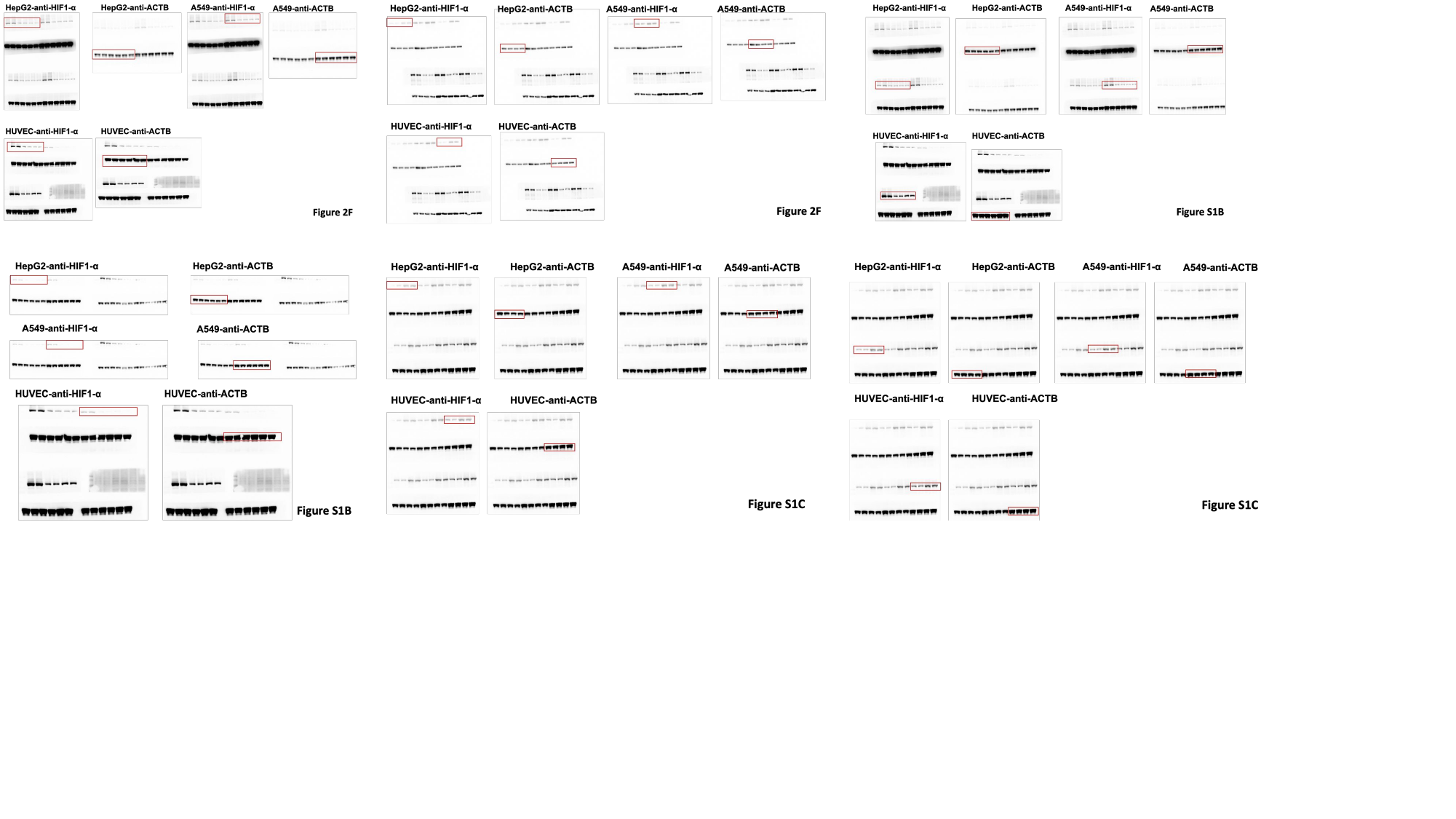

## Slide 2
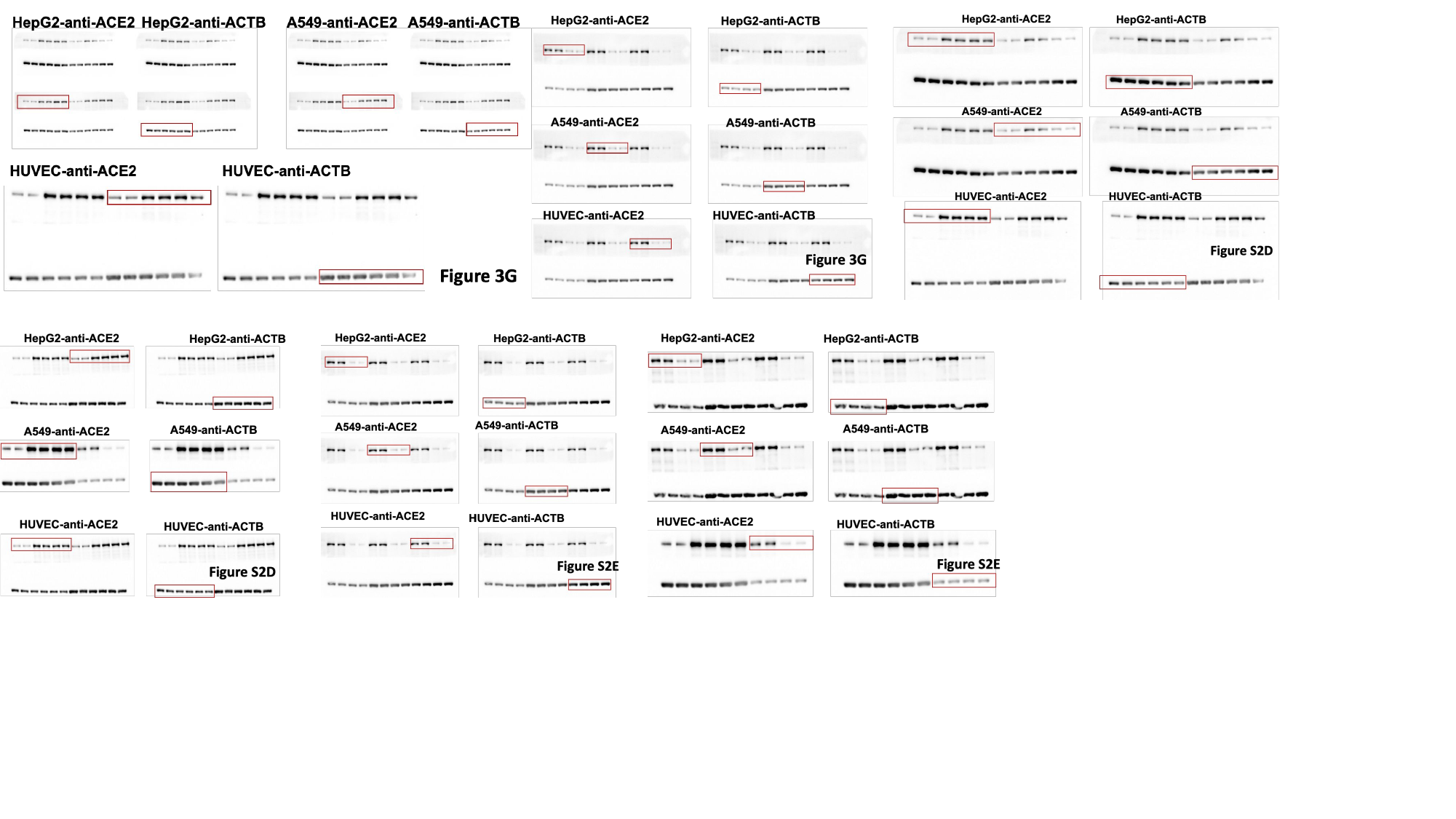

## Slide 3
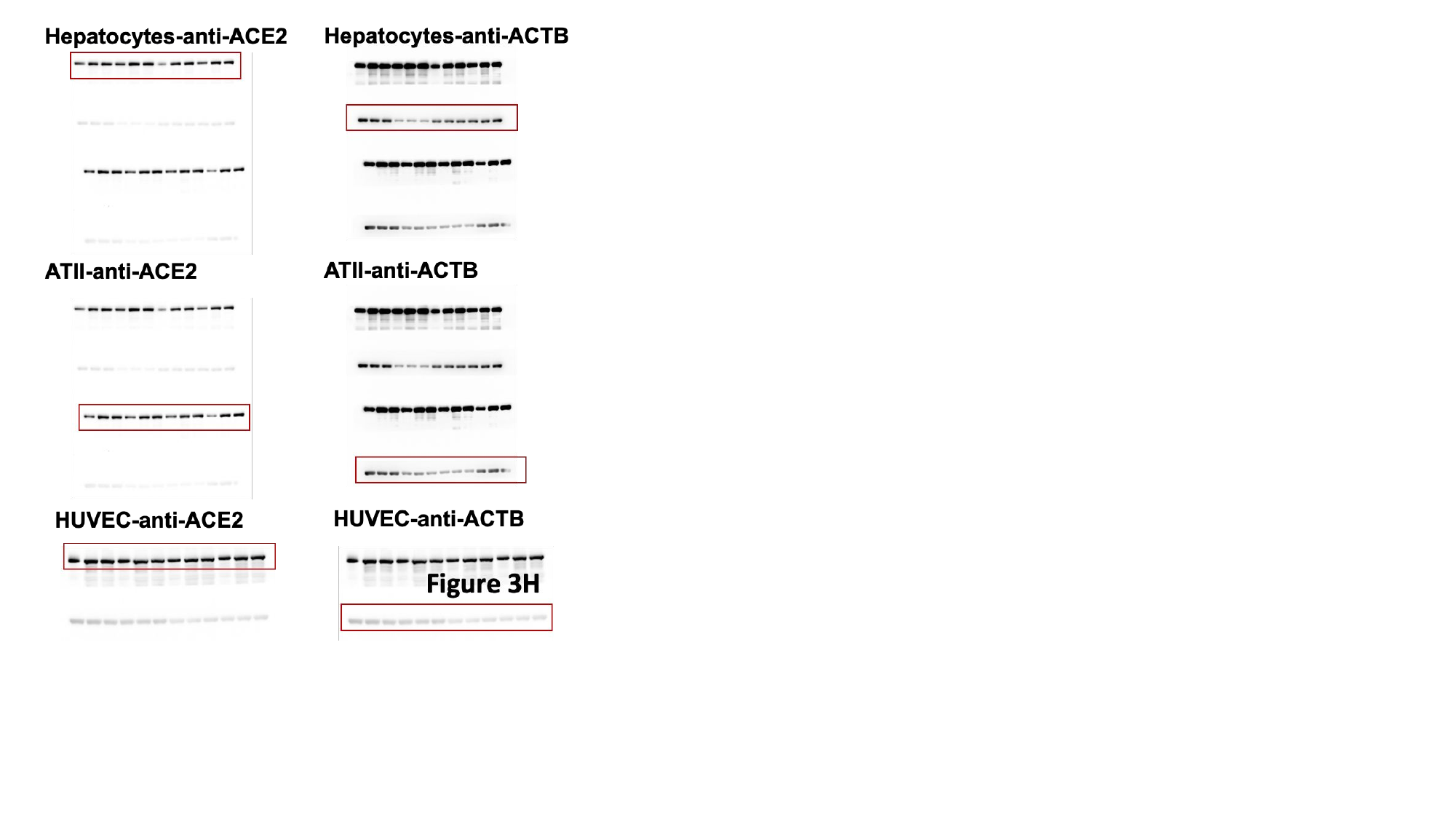

## Slide 4
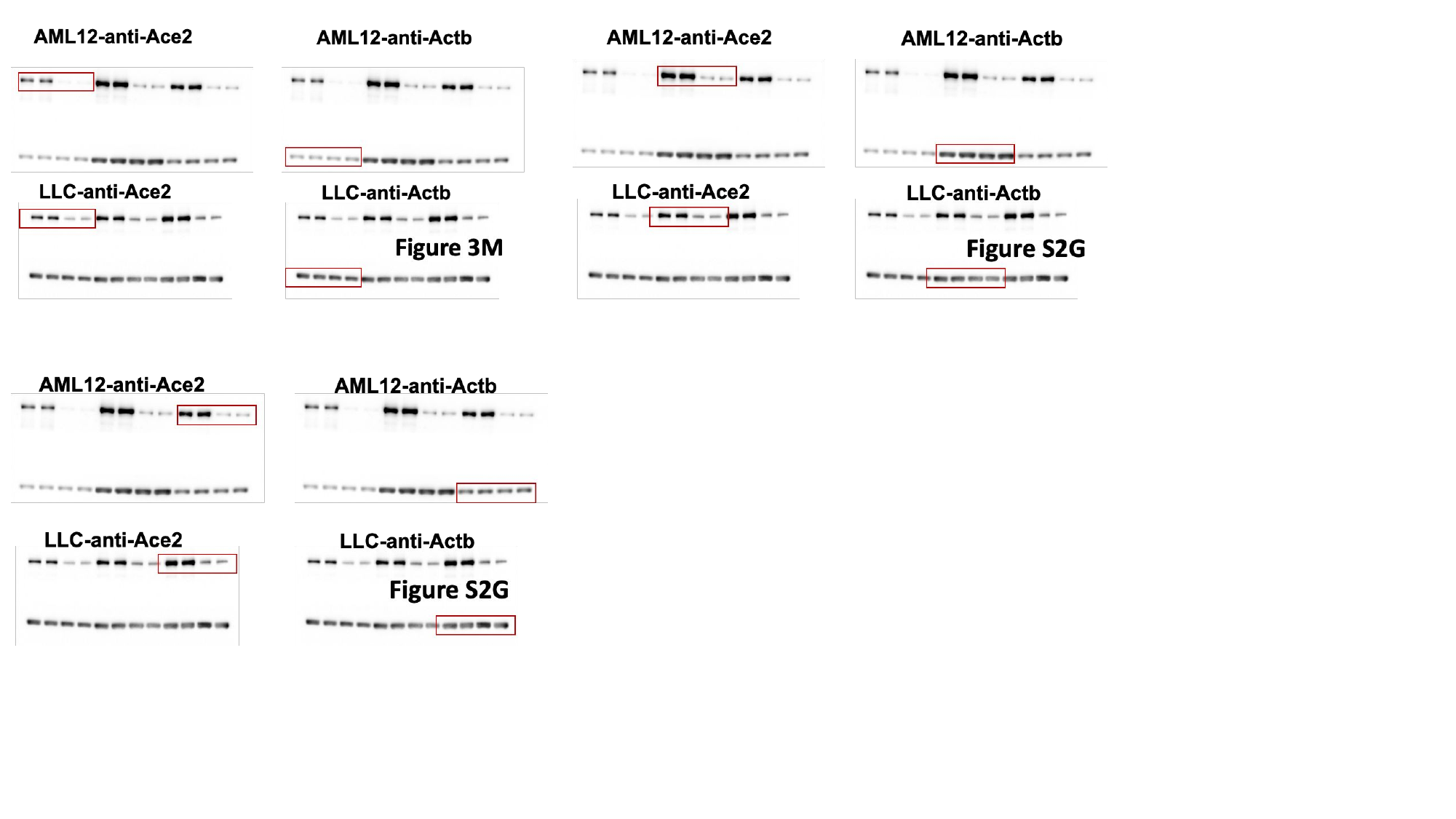

## Slide 5
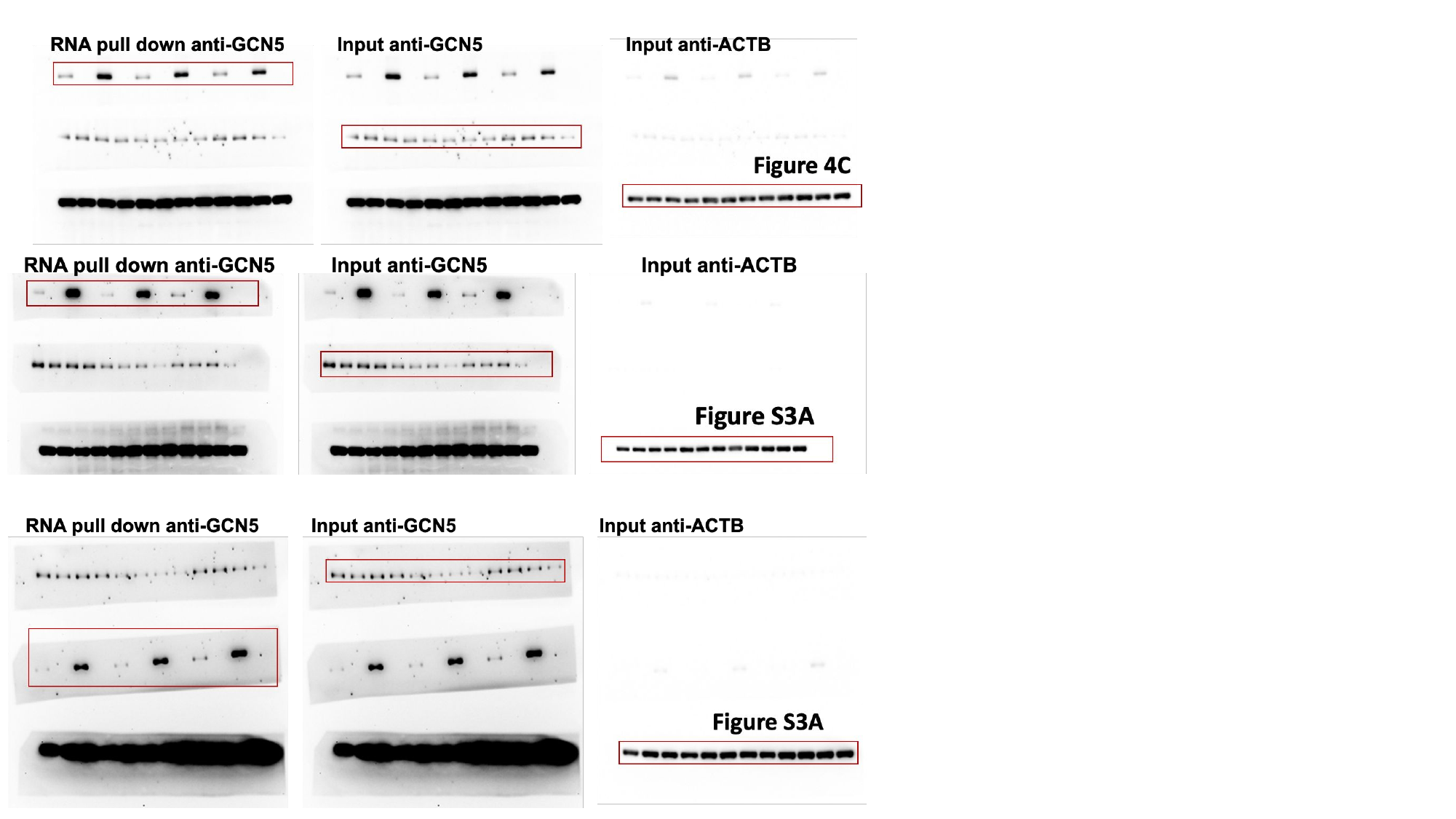

## Slide 6
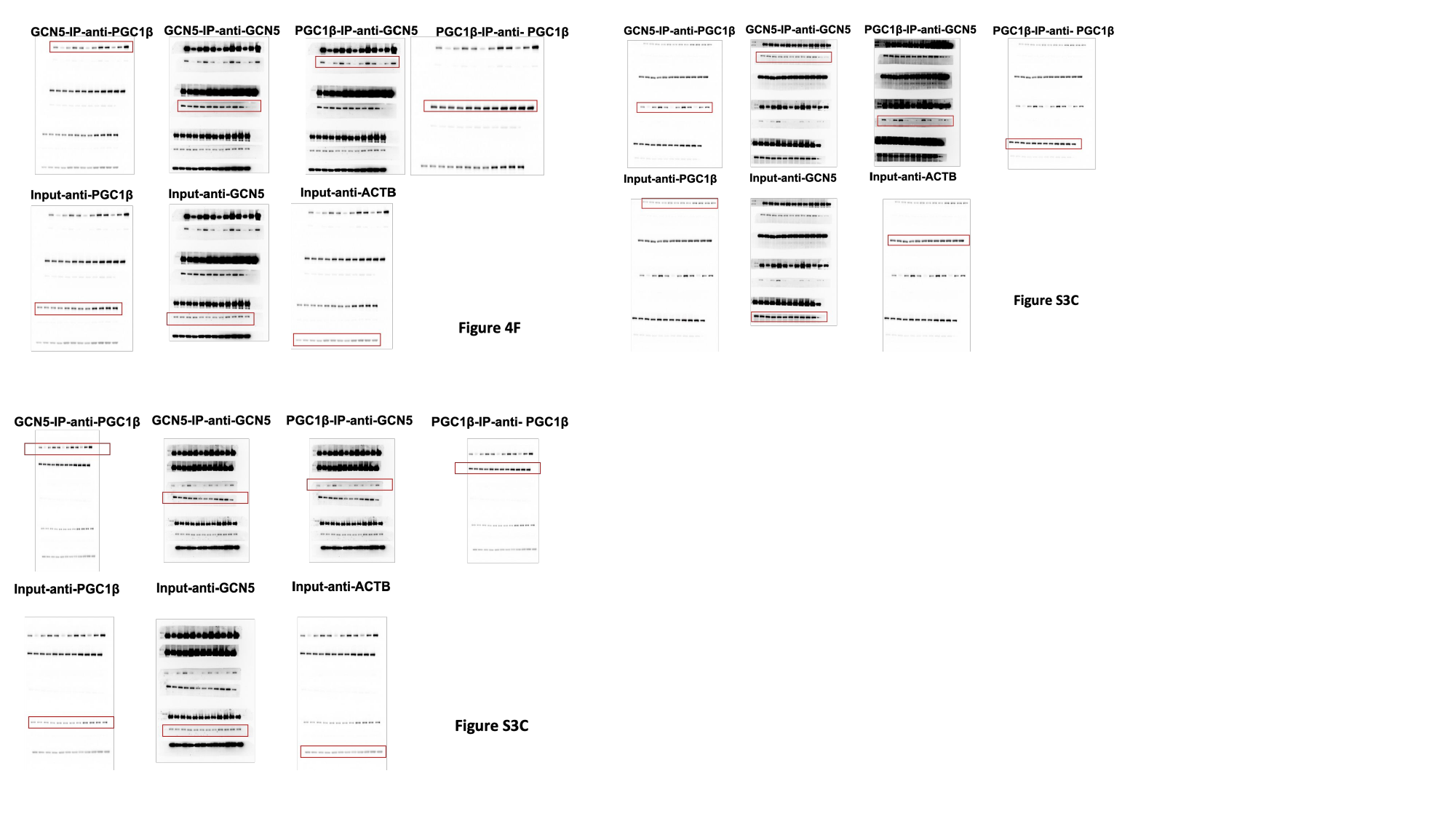

## Slide 7
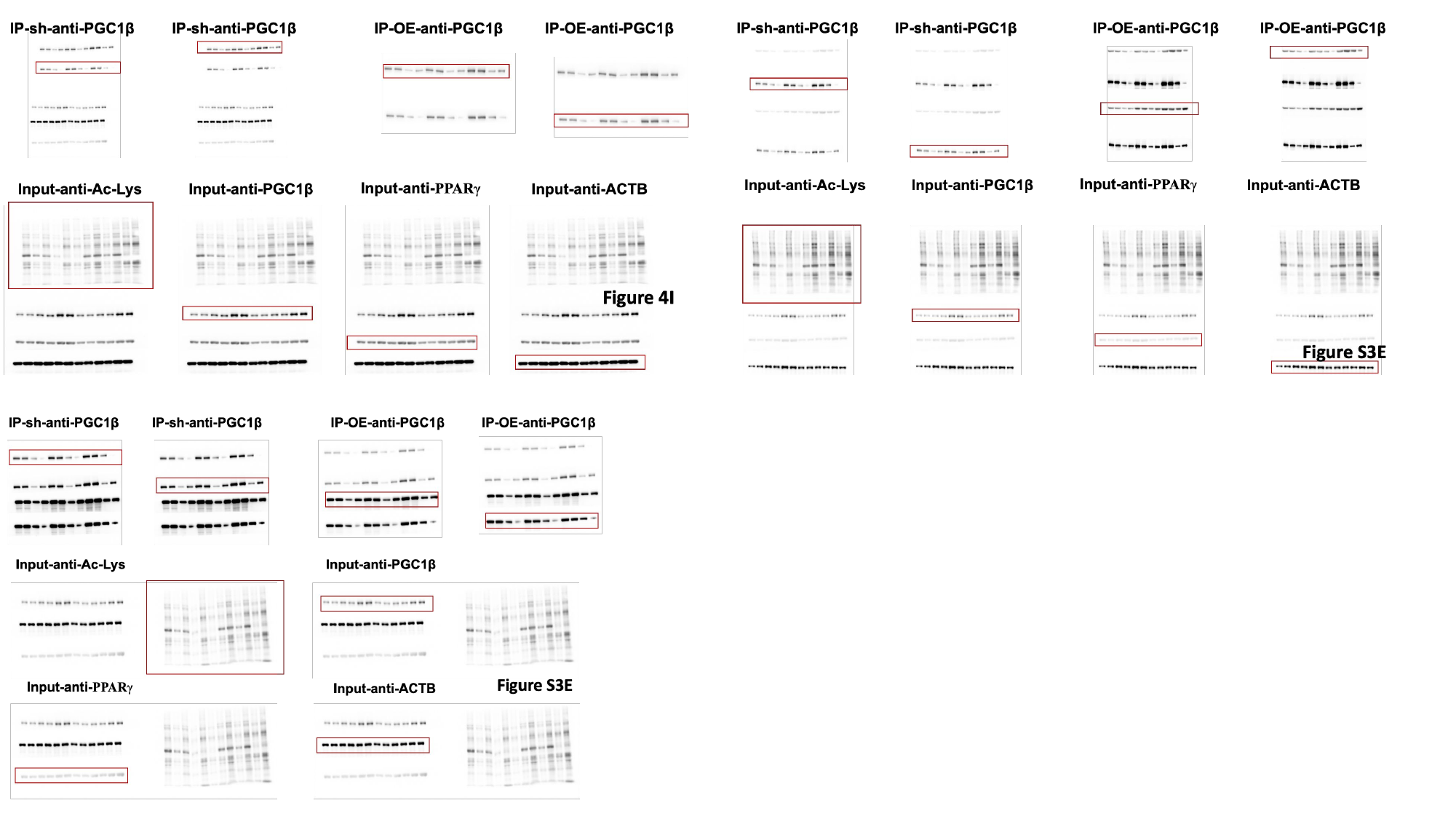

## Slide 8
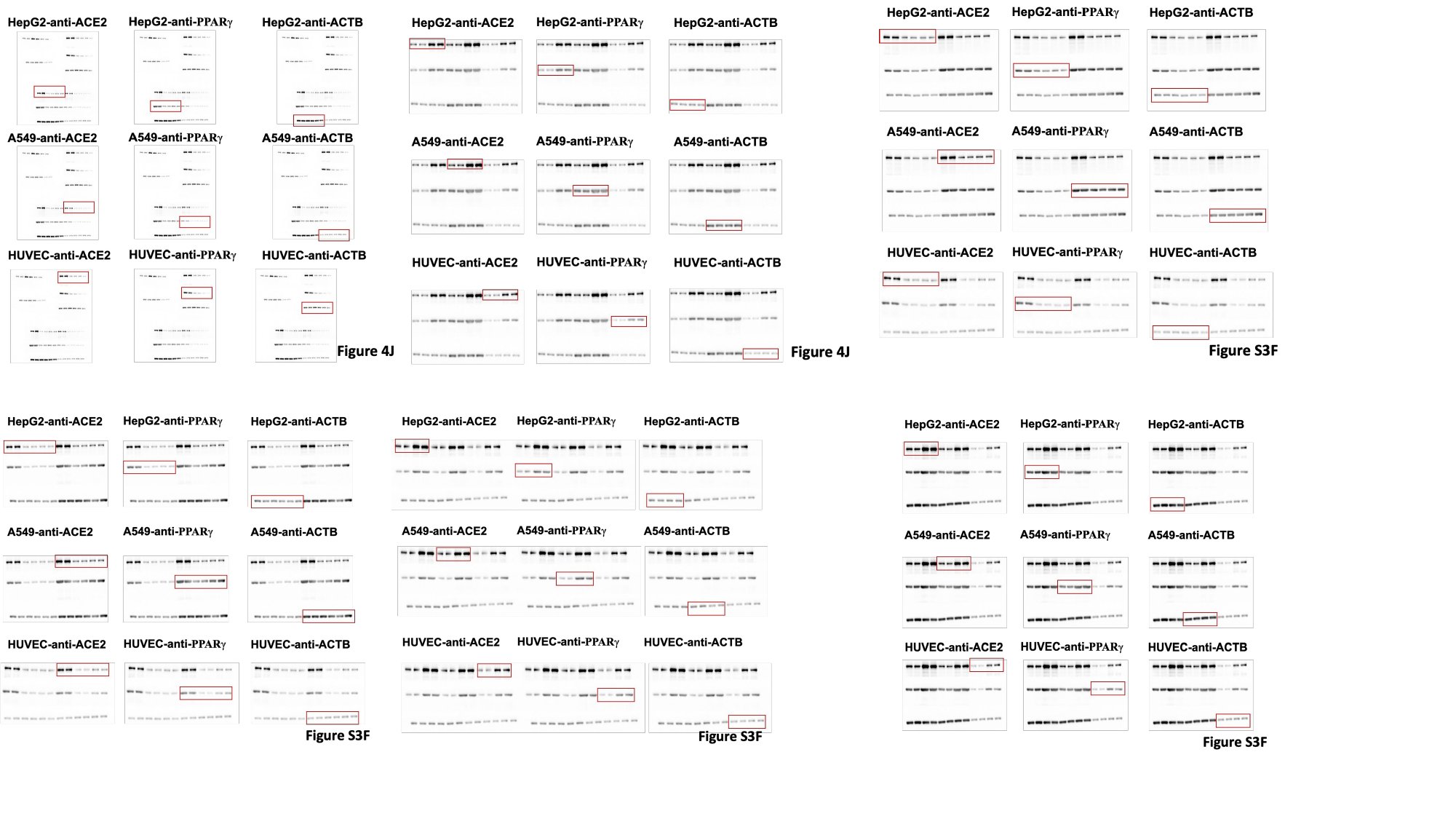

## Slide 9
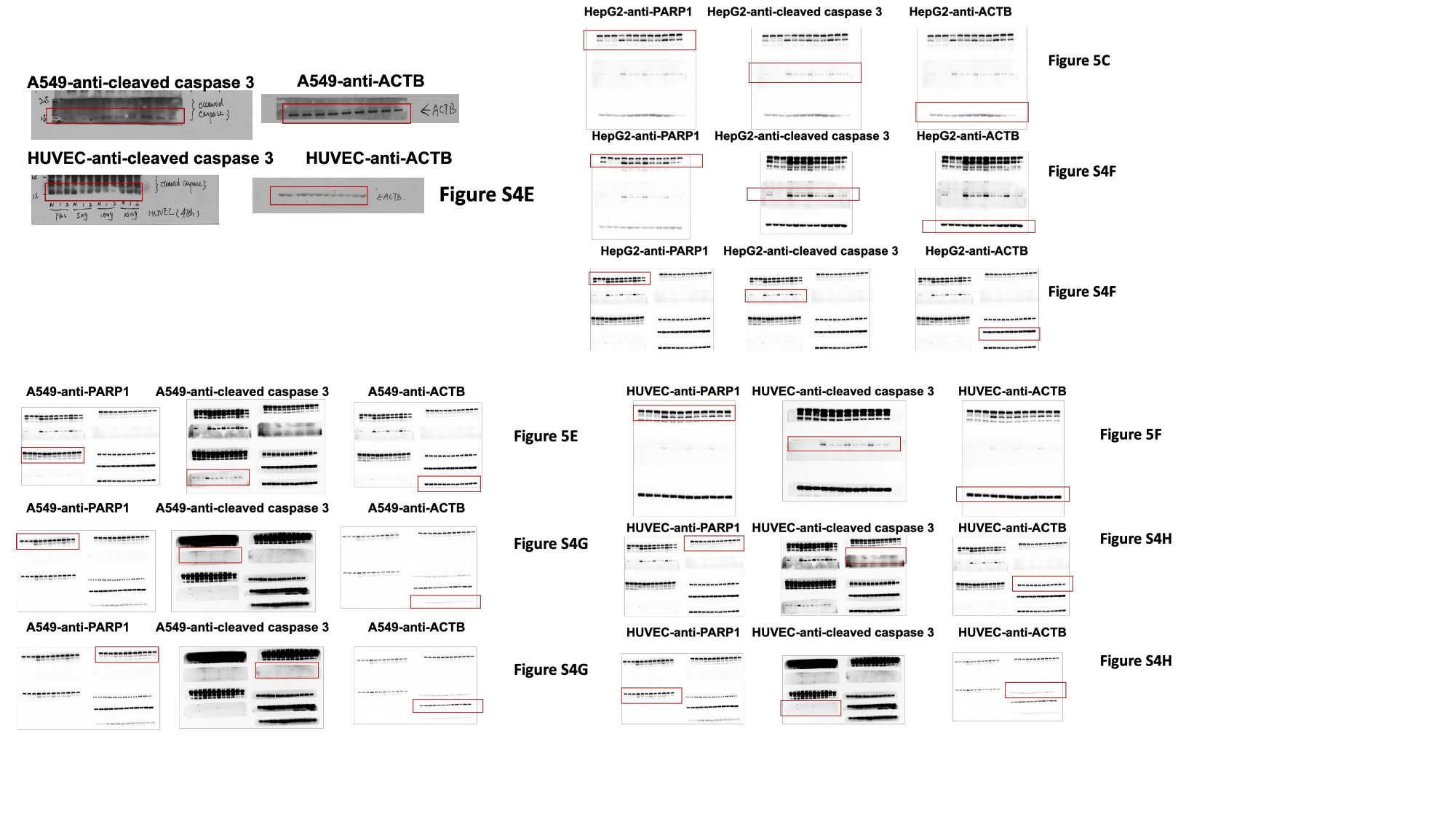

## Slide 10
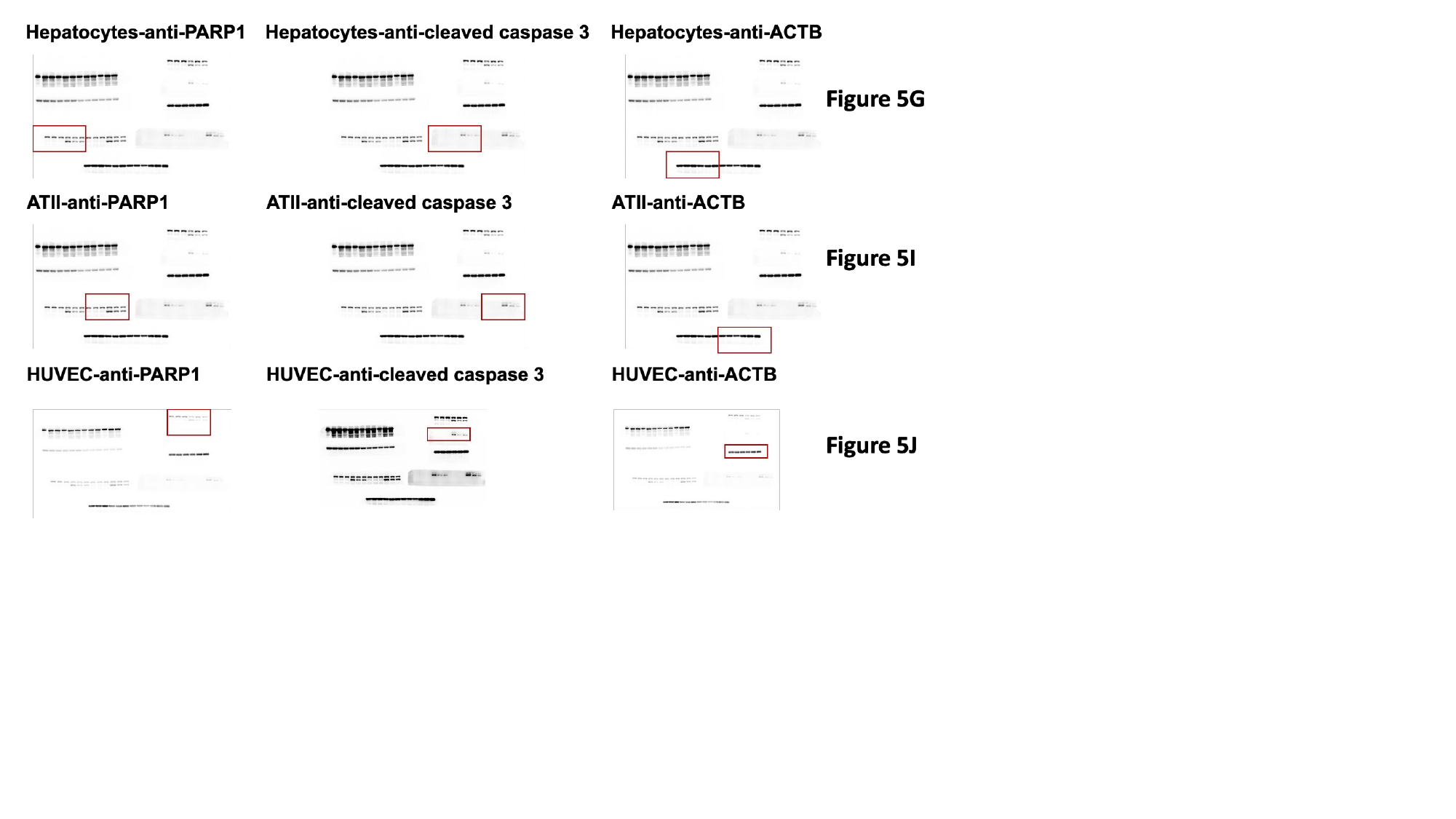

## Slide 11
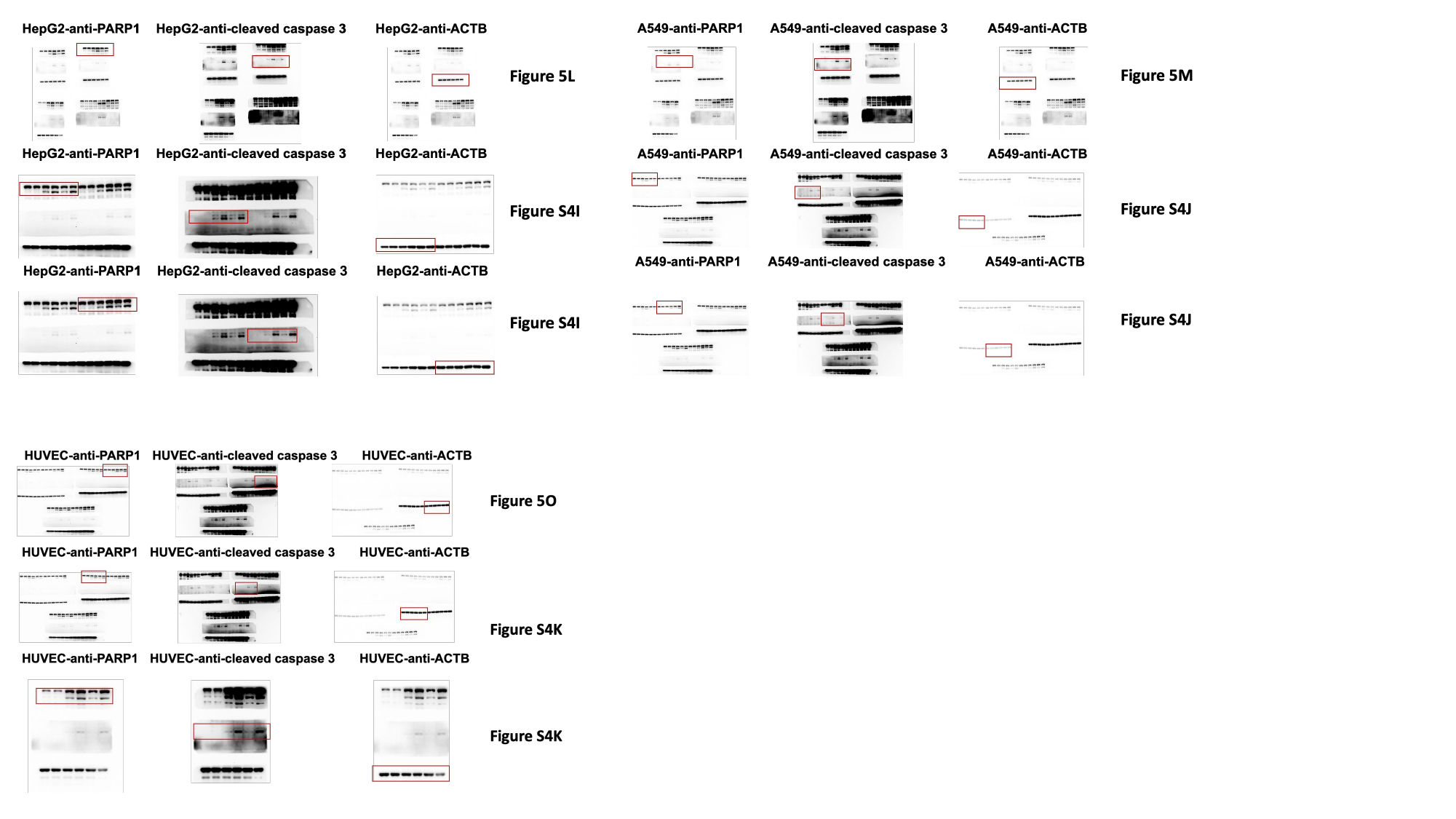

Supplement: Supporting Information 4 [file mmc4.pptx]
